# The SEE-IT Trial: emergency medical services **S**treaming **E**nabled **E**valuation **I**n **T**rauma: study protocol for an interventional feasibility randomised controlled trial

**DOI:** 10.1136/bmjopen-2023-072877

**Published:** 2023-04-24

**Authors:** Lucie Ollis, Simon S Skene, Julia Williams, Richard Lyon, Cath Taylor, the SEE-IT Trial Group, Kate Bennett-Eastley

**Affiliations:** 1 School of Health Sciences, University of Surrey, Guildford, Surrey, UK; 2 Surrey Clinical Trials Unit, University of Surrey, Guildford, UK; 3 South East Coast Ambulance Service NHS Foundation Trust, Banstead, Surrey, UK; 4 University of Hertfordshire School of Health and Social Work, Hatfield, UK; 5 Kent, Surrey & Sussex Air Ambulance, Redhill, UK

**Keywords:** accident & emergency medicine, telemedicine, information technology, feasibility studies, primary care, trauma management

## Abstract

**Introduction:**

Accurate and timely dispatch of emergency medical services (EMS) is vital due to limited resources and patients’ risk of mortality and morbidity increasing with time. Currently, most UK emergency operations centres (EOCs) rely on audio calls and accurate descriptions of the incident and patients’ injuries from lay 999 callers. If dispatchers in the EOCs could see the scene via live video streaming from the caller’s smartphone, this may enhance their decision making and enable quicker and more accurate dispatch of EMS. The main aim of this feasibility randomised controlled trial (RCT) is to assess the feasibility of conducting a definitive RCT to assess the clinical and cost effectiveness of using live streaming to improve targeting of EMS.

**Methods and analysis:**

The SEE-IT Trial is a feasibility RCT with a nested process evaluation. The study also has two observational substudies: (1) in an EOC that routinely uses live streaming to assess the acceptability and feasibility of live streaming in a diverse inner-city population and (2) in an EOC that does not currently use live streaming to act as a comparator site regarding the psychological well-being of EOC staff using versus not using live streaming.

**Ethics and dissemination:**

The study was approved by the Health Research Authority on 23 March 2022 (ref: 21/LO/0912), which included NHS Confidentiality Advisory Group approval received on 22 March 2022 (ref: 22/CAG/0003). This manuscript refers to V.0.8 of the protocol (7 November 2022). The trial is registered with the ISRCTN (ISRCTN11449333). The first participant was recruited on 18 June 2022.

The main output of this feasibility trial will be the knowledge gained to help inform the development of a large multicentre RCT to evaluate the clinical and cost effectiveness of the use of live streaming to aid EMS dispatch for trauma incidents.

**Trial registration number:**

ISRCTN11449333.

**Strengths and limitations of this study**

- The mixed methods approach to evaluation of feasibility and acceptability uses a combination of routinely collected data, observational data, interviews and survey methods.
- A comprehensive process evaluation will be undertaken to understand acceptability and impact of using live streaming from the perspective of 999 callers and emergency operations staff.
- The design (testing for a week each month over 6 months) builds consideration of seasonal variation in trauma into the design, while enabling time to adapt and improve the protocol as the SEE-IT Trial progresses.
- Recruitment of 999 callers may prove challenging due to the nature of their involvement in incidents; and investigating any impact of wider diversity factors is dependent on the characteristics of callers during the trial periods and we may not have sufficient data for this. The Ambulance Trust where live streaming will be tested uses NHS Pathways (as used in half of Ambulance Trusts in the UK); the SEE-IT Trial will give us insight into how live streaming would work with this dispatch system but may not extrapolate fully to areas that use other systems for triage/decision support.

## Introduction

### Background and rationale

Emergency medical services (EMS) provide emergency medical care at the scene of incidents before patients are conveyed to hospital (eg, blood transfusions and emergency anaesthesia). To save lives and prevent long-term disability, timely and accurate dispatch of pre-hospital resources is critical.^1^ Evidence suggests trauma patients should receive definitive care within 1 hour of the incident (The ‘Golden Hour’), otherwise the risk of morbidity and mortality increases significantly.^2^ Where severe injury is suspected by EMS (eg, major trauma), specialist paramedics in critical care and/or helicopter EMS (HEMS) may be dispatched to the scene to care for the patient and/or convey the patient to hospital.

When a 999 call is made in the UK, the dispatchers in the emergency operations centre (EOC) need to decide which resources to send to the scene by gathering information about what has happened and the clinical state of the patient(s). The dispatchers rely on 999 callers and other bystanders to accurately relay essential information.^3^ However, lay public 999 callers can sometimes provide inaccurate information due to language barriers, subjectivity, the emotional impact of being at the scene and limited medical training or knowledge.^3 4^ Dispatch response is often recognised as a weak link in the EMS chain,^5^ often involving either under-resourcing or over-resourcing.^6–9^ Previous studies have shown that up to 50% of HEMS (air ambulance) dispatch and 25% of road ambulance dispatch for suspected trauma patients are not appropriate.^6–9^

It is vital that EMS are being dispatched to incidents where they are needed due to finite and often limited resources. If dispatchers were able to see the patient and scene via live video streaming through a 999 callers’ smartphone camera, it could be easier to make more accurate and timely decisions about dispatch of EMS.^4^ There is evidence that video-assisted cardiopulmonary resuscitation (CPR) has the potential to improve patient outcomes,^10–16^ but there is a lack of research evidence to support the use of live video streaming for EMS dispatch for trauma.

NHS policies and the Department of Health and Social Care actively encourage the use of innovative technologies to improve patient and healthcare outcomes.^17^ The benefits of using video in other healthcare settings are growing, for example, for remote healthcare consultations,^18–21^ but evidence is sparse in relation to use in pre-hospital settings, despite being used routinely in some ambulance services. There is some limited evidence of the benefits and acceptability of live streaming to aid HEMS dispatch,^4^ but its impact on clinical or economic outcomes has not yet been evaluated. Also, the psychological impact of dispatchers viewing the scene and impact on 999 callers has not been investigated for exposure to trauma.

### Objectives and research questions

Main research question: is it feasible to conduct a future randomised controlled trial (RCT) to assess the clinical and cost effectiveness of using GoodSAM live video streaming to improve targeting of emergency medical resources? The overall aim of this research is to assess the feasibility of implementing and evaluating GoodSAM live streaming in a definitive RCT.

The objectives are:

1. To obtain data required to inform the design of a subsequent RCT (eg, event rate, screening rate, effect size/precision for outcomes health economic data).
2. To test trial processes including randomisation and data collection methods.
3. To conduct a nested process evaluation to test the acceptability and feasibility of using GoodSAM live streaming from provider and public perspectives (eg, training, video feasibility, video acceptability, psychological harm to 999 callers and/or dispatch staff).

Further research questions relating to these objectives can be found in the study protocol published on the National Institute for Health and Care Research (NIHR) study webpage.^22^

## Methods and analysis

### Trial design and study setting

The SEE-IT Trial is a feasibility RCT with a nested process evaluation in an EOC in South East England (South East Coast Ambulance Service NHS Trust). This study includes two observational substudies (1) in an EOC that routinely uses live streaming to assess acceptability and feasibility of use of live streaming in a diverse inner-city population (London Ambulance Service NHS Trust) and (2) in an EOC that does not currently use live streaming to act as a comparator site regarding the psychological well-being of EOC staff using versus not using live streaming (East of England Ambulance Service NHS Trust). In addition, 11 NHS hospital trusts covered by the main trial site will be involved in identifying and consenting patients for access to their medical records including: University Hospital Southampton NHS Foundation Trust; St George’s University Hospitals NHS Foundation Trust; Medway NHS Foundation Trust; Surrey and Sussex Healthcare NHS Trust; Maidstone and Tunbridge Wells NHS Trust; East Sussex Healthcare NHS Trust; East Kent Hospitals University NHS Foundation Trust; Dartford and Gravesham NHS Trust; King’s College University Hospital NHS Trust; Royal Surrey County Hospital NHS Foundation Trust and University Hospitals Sussex NHS Foundation Trust.

The study was planned to run for 18 months from 1 October 2021 to 31 March 2023. Pressures on the NHS and ambulance services meant the start of the trial period was delayed 4 months to June 2022. Therefore, the overall study end date is now 31 July 2023. There were nine amendments made to the protocol during the feasibility study, outlined in the protocol on the NIHR study webpage.^22^

### Eligibility criteria

This study includes three types of participants; trauma patients, lay public 999 callers and EOC staff. See tables 1 and 2 for inclusion and exclusion criteria, respectively.

**Table 1:** Participant inclusion criteria

Participant type	Main feasibility trial	Inner-city observational substudy	Staff well-being substudy
Trauma patients	All trauma patients during the six trial observation weeks who are the subject of 999 calls involving major trauma, judged by the HEMS dispatcher and/or critical care paramedics (CCPs) as likely to require enhanced dispatch.	All trauma patients during observed shifts that involve trauma and are screened by HEMS dispatchers or critical care advanced paramedic practitioner dispatchers (APPs) who attempt to use GoodSAM during the call.	N/A
Lay public 999 callers	999 callers during the six trial weeks where the incident involves major trauma (defined as per above).	All 999 callers during observed shifts that involve trauma and are screened by HEMS dispatchers or critical care APPs who attempt to use GoodSAM during the call.	N/A
EOC staff	All CCPs, HEMS dispatchers and research paramedics.	All HEMS dispatchers and critical care APPs.	All CCPs and HEMS dispatchers.

CCPs in the main feasibility trial site and APPs in the inner-city observational substudy are the ways different Ambulance Trusts describe specialist paramedics in critical care.

EOC, emergency operations centre; HEMS, Helicopter emergency medical services.

**Table 2:** Participant exclusion criteria

Participant type	Main feasibility trial	Inner-city observational substudy	Staff well-being substudy
Trauma patients	Any emergencies of a suspected medical origin (eg, heart attack or stroke).	Any emergencies of a suspected medical origin (eg, heart attack or stroke).	N/A
Lay public 999 callers	Calls will be excluded where: (1) 999 caller is not at the scene; (2) 999 call originates from a landline; (3) 999 call originates from another emergency service, for example, police or fire; (4) 999 calls where resource (excluding community first responder, CFR) will arrive on scene before live streaming could be activated; (5) 999 call ended before transfer for activation of live streaming; (6) 999 calls where another incident takes priority and (7) calls where clinical acuity is found to be lower than the threshold for the study (not major trauma). All callers identified by the dispatchers as a child caller (under 16 years old) and those who select they are under 16 on the 999-caller survey will be excluded.	All callers identified by the dispatchers as a child caller (under 16 years old) and those who select they are under 16 on the 999-caller survey will be excluded.	N/A
EOC staff	EOC staff not mentioned in the inclusion criteria.	EOC staff not mentioned in the inclusion criteria.	EOC staff not mentioned in the inclusion criteria.

EOC, emergency operations centre.

### Intervention and randomisation

999 calls in the main trial site during the six observation weeks (up to 42 days; 84 shifts) will be randomised 1:1 to working shift intervention or standard care. Control shifts will follow standard care ambulance dispatch protocol with a 999 caller using a telephone (voice only) and the call taker using NHS Pathways ambulance dispatch tool.

In the intervention arm, a live streaming technology called GoodSAM Instant-on-Scene^23^ will be tested. The technology allows a 999 caller to stream live video footage from the scene of an incident to the EOC. 999 calls allocated to intervention will initially follow the standard NHS Pathways dispatch protocol until ambulance dispatch prioritisation has been determined. EMS will be dispatched as normal, without delay. The call handler in the EOC will then transfer the caller to the HEMS dispatcher or critical care paramedic (CCP) and live streaming will be activated. EMS resource allocation may be adjusted following this.

If at any point the dispatcher feels they do not want to view the scene (eg, due to psychological impact of viewing the scene) or it is not safe to continue, the 999 caller will be thanked for their help and the call will be ended. The dispatchers will be provided with training and guides outlining the trial processes to ensure adherence to intervention and study protocols.

### Outcomes

#### Primary outcomes

The primary outcome is the decision regarding the feasibility of undertaking a definitive RCT based primarily on meeting the specified progression criteria (see table 3).

**Table 3:** Progression criteria from feasibility RCT to future RCT

GREEN; proceed to definitive study—GO	AMBER; consider protocol amendments to improve criteria	RED; do not proceed to main trial—STOP
≥70% of callers with smartphones agreeing and able to activate live streaming	≥50% of callers with smartphones agreeing and able to activate live streaming	<50% of callers with smartphones agreeing and able to activate live streaming
≥50% of requests to activate live streaming resulting in footage being viewed	≥30% but <50% of requests to activate live streaming resulting in footage being viewed	<30% of requests to activate live streaming resulting in footage being viewed
Air Ambulance (HEMS) stand-down rate reducing by ≥10% and/or change in dispatch decision as a result of live streamed footage confirmed as being appropriate in ≥10% cases	Air Ambulance stand-down rate reducing by ≥5% and/or change in dispatch decision as a result of live streamed footage confirmed as being appropriate in ≥5% cases	No change in Air Ambulance stand-down rate and/or change in dispatch decision as a result of live streamed footage
Rates of psychological harm (based on the survey measures) not significantly greater in 999 callers using live streaming compared with those not; and no significant difference in change to psychological harm over time in staff (CCPs, HEMS dispatchers, research paramedics) compared with change in staff in a comparison EOC not using live streaming/streamed from scene footage	–	Evidence of significantly greater harm in either 999 callers or EOC staff using live streaming compared with EOC not using live streaming.

CCP, critical care paramedic; EOC, emergency operations centre; HEMS, helicopter emergency medical services; RCT, randomised controlled trial.

In addition to the quantitative progression criteria described in table 3, the research team will consider qualitative data collected as part of this study (eg, interviews, observations and free text questions in surveys) when reviewing progression to a definitive RCT.

#### Secondary outcomes

Speed of appropriate EMS dispatch: speed will be measured using time-stamped data from start of 999 calls to appropriate deployment with judgement of ‘appropriateness’ to be based on algorithms developed through expert consensus (see below) and using medical records data up to 3 months post-incident where consent is provided; stand-down rate (de-escalation); missed jobs (eg, not prioritised for HEMS/CCP despatch, either due to lack of resource or inappropriate prioritisation); requests for further ambulance resources from scene. Psychological harm will be assessed using two validated scales^24 25^ pre-trial and post-trial in 999 callers from both arms and staff viewing the footage (including staff in comparison EOC).

The appropriateness of dispatch will be determined based on algorithms developed through expert consensus before the trial starts. Appropriateness is defined as the need for intervention and/or transport by the response vehicle/staff dispatched to scene (eg, for HEMS dispatch that a critical care intervention and/or transportation by helicopter was required^9^). A binary outcome decision tree will be developed with the reasons for appropriate/inappropriate dispatch categorised and agreed (inappropriate dispatch will be further categorised as being ‘over’ or ‘under’ resourced).

### Participant timeline

There will be six trial weeks, 1 week a month from June to November 2022. Each trial week will include up to 14 randomised shifts (day and night). EOC staff in the main trial site and staff well-being substudy will be invited to participate in the staff survey pre-trial and post-trial period. Staff in the main trial site will be invited to partake in interviews through the survey. 999 callers in the main trial site will be sent a text message after the eligible incident inviting them to participate in a survey 6–8 weeks post incident and a subsequent interview. Patients will be approached for consent for their medical records to be accessed up to 3 months after the incident has occurred.

In the observational substudy, EOC staff will be observed using GoodSAM live streaming over a 3-month period and invited to partake in interviews. 999 callers will be invited to partake in the survey within 1 week of the incident they called 999 for and subsequently invited to partake in an interview about their experiences of using GoodSAM live streaming.

### Sample size

We estimate there will be approximately 250 trauma incidents (including 300 patients) over the six observation weeks (125 allocated to intervention), which will allow for an estimate of true event rate within precision of ±0.75 events per day; and allow estimation of speed to appropriate response with an SE of <5%. These sample size estimations are based on convenience sampling over the six defined trial observation weeks. We estimate there will be approximately 350 lay public 999 callers (250 from the main trial site and 100 from the observational substudy) and up to 202 EOC staff (86 from the main trial site, 30 from the observational substudy and 86 from the third EOC).

### Recruitment: identification and consent

#### Trauma patients

Trauma patients will be identified by research paramedics (RPs), using the eligibility criteria. RPs will observe all trial shifts. For eligible calls in the main trial site, the computer-aided dispatch (CAD) number(s) relating to the incident will be recorded by the RP as the identifier to share with the conveying hospital to approach for consent. Informed consent will be sought from either the patient, their consultee or the patient’s parent or guardian (either in the hospital before discharge or via telephone, email or post) in all hospital trusts included in the trial (trauma units and major trauma centres covered by the EOC). RPs may consent patients in person with assistance from the hospital to identify the patient(s). Approval has been granted by the Confidentiality Advisory Group (Section 251 application) to access medical records for casualties who die before consent can be sought (and where there is not a power of attorney in place for that individual). National data opt-out status will be checked for all deceased patients.

#### 999 callers

In the main feasibility site, the RP will determine eligibility of the 999 callers and prompt the dispatchers to send a text message via the CAD, inviting the 999 caller to participate in a survey about their experience of calling 999 for the incident. The text message will include a link to the participant information sheet (PIS), consent form and contact details for the research team. A reminder text message will be sent to 999 callers within 1 week of the incident. Those who participate in the survey will be invited to partake in an interview and selected to represent a range in demographics (age, ethnicity, gender), type of incident they witnessed and evidence or not of psychological harm from survey responses.

In the inner-city observational substudy, at the end of the call (once resources have been dispatched) 999 callers will be asked for permission to store their name and number to share with the researcher to be invited to participate in the study. Those who consent will initially be contacted by phone so the researcher can explain the study and ask their permission to send them further details about the study via text message or email. The researcher will then send a text message or email with a link to the PIS and consent form, which once completed will re-direct to the survey. Reminder texts/emails will be sent up to 1 week after the incident. Only those who participate in the survey will be invited to partake in an interview.

#### EOC staff

In the main trial site, the use of live streaming has been approved at organisation level and thereby staff are not individually consented to use live streaming but instead will be given the opportunity to opt out of trial shifts. They will otherwise be required to follow organisational protocols if they are working on a trial shift. In the observational substudy, HEMS dispatchers and critical care APPs will consent on an individual basis to being observed using live streaming. The researcher will contact staff to invite them to participate in an interview about the use of live streaming in their organisation. The local PI at both EOCs participating in the staff survey will share the staff survey link with all eligible staff pre-trial. All staff who enter their email address to complete the post-trial survey will be emailed a link to the survey post-trial. Those who participate in the staff survey at the main trial site will be invited to partake in an interview about their views and experiences of live streaming; selected to include different types of staff, for example, age, gender and experience.

### Assignment of intervention

Working shifts are randomised 1:1 to intervention (GoodSAM) or standard ambulance care using a minimisation algorithm to ensure balance between weekday/weekend (Mon–Thurs vs Fri–Sun) and daytime/night-time shifts (06:00 to 18:00 vs 18:00 to 06:00).

A small number of shifts in the final observation week will be used to test the feasibility of individual call randomisation using a pre-prepared randomisation list. All randomisations will be undertaken by a statistician at Surrey Clincial Trials Unit to ensure the allocation is independent of the research team.

### Data collection methods

#### Observations of EOC processes

An experienced researcher will observe EOC processes, decision making processes, fidelity to the protocol and impact of contextual variability (eg, time of day, weather) during the trial weeks in the main trial site (3–6 hours for 3 days in each observation week). In the observational substudy, a second experienced researcher will observe up to 24 shifts over a 3- month period. Observational data will be recorded using fieldnotes capturing both descriptive and conceptual observations/interpretations.

The observational and interview data collection and analysis will be underpinned by theories informing the proposed mechanisms of effect, for example, situational awareness (SA)^26^; decision making (DM)^27^; the implementation of a new technology (TAM)^28–30^ and Consolidated Framework for Implementation Research.^31^ Events will be observed in real time and the use of live streaming will be understood in the context of how the use of visual technology impacts on staff assessment of situations and action taken (SA and DM), as well as the factors influencing how people relate to the introduction of a new technology (TAM). Equally, in situations where live streaming is not used, theories of SA and DM will inform observations in context.

#### Decision making data and clinical data from trauma patients

During the trial weeks, the RPs will collect data live during incidents including the CAD number; information about decision making; the patients’ name, age and sex (if available); details of the trauma incident (eg, single/multiple casualties; general nature of incident, eg, road traffic collision/assault); dispatch decisions and the use of live streaming for example, quality of sound/picture. This information will be recorded on a piloted, study-specific proforma completed at the time of the incident. Further details about EMS dispatch (including speed to appropriate dispatch) and time stamped data for each incident will be accessed by RPs after the incident.

Following consent to access medical records (process described in recruitment section), the RPs will extract relevant information from patients’ medical records about the injuries sustained and the treatments received (up to 3 months post-incident). Using the expert panel algorithms, the RPs will rate the appropriateness of dispatch. A random 10% sample of incidents from intervention and control calls will be independently reviewed by the expert panel using the same data, to assess reliability.

#### 999 callers

999 callers in the main trial site will be invited to complete surveys via Qualtrics online survey software^32^ 6–8 weeks post incident to assess psychological harm and semistructured interviews regarding experiences and acceptability of the use of live streaming. The questionnaire will include demographic questions, The Impact of Events Scale—Revised,^24^ The General Health Questionnaire-12 item version^25^ and questions about acceptability and experience of live streaming.

In the inner-city observational substudy, 999 callers will be invited to complete surveys via Qualtrics^32^ up to 1 week after the incident. The questionnaire will include a demographic questionnaire and questions about their experiences of using live streaming during their recent 999 call for example, ease of use, usefulness. Those who take part in the questionnaire will be invited to participate in an interview regarding experiences and acceptability of using live streaming.

999 callers at both sites who participate in the survey will be offered the choice of a £10 shopping voucher or £10 donation to the local air ambulance charity. A second voucher or donation will be offered to those who complete an interview.

#### EOC staff survey and interviews

Before the trial starts, EOC staff at the main trial site and staff well-being substudy will be emailed by the local principal investigator (PI) including the PIS, consent form and a link to the online survey via Qualtrics.^32^ The survey will include a demographic questionnaire, The Impact of Events Scale—Revised,^24^ The General Health Questionnaire-12 item version^25^ and questions about live streaming. Those who complete both pre-trial and post-trial questionnaires will be entered into a prize draw for one of five £50 retail vouchers (five available at each site). In the main trial site, approximately 12–18 staff will be invited to participate in an interview and will be paid overtime rates for their participation. Staff in the observational substudy who take part in an interview will be compensated for their time with a £45 Amazon voucher. The different methods for reimbursing time have been requested by the respective organisations.

### Data management

A detailed data management plan has been produced outlining the processes for completion, transfer and storage of study data in line with the Ambulance Trust and University policies, the requirements of the Sponsor and General Data Protection Regulation (GDPR). The data management plan will be shared with all those responsible for data collection on the trial and will form the basis of a Data Protection Impact Assessment and data processing agreements between the parties.

### Statistical methods and data analysis

#### Quantitative analysis

A full statistical analysis plan (SAP) will be reviewed by key members of the research team and approved by the steering committee before analysis is undertaken.

As this is a feasibility study, statistical analysis will focus on providing estimates and confidence intervals of key rates such as the number of eligible calls per day, uptake of video intervention and timing and appropriateness of dispatch to inform a subsequent RCT. Outcome measure will be summarised by arm and estimates reported with confidence intervals to inform future sample size calculations.

The SAP will outline any expected exploratory analysis of outcomes that may be useful to inform a subsequent RCT including any signal of potential efficacy, but it is noted that the study is not powered for this and so any interpretation would be limited to the direction and magnitude of any effect. Missing data (including outcome data) will be summarised, but all observed data will be included in any analysis according to the randomised allocation following the intention-to-treat principle.

#### Qualitative analysis

The process evaluation will include analysis of live streaming usage data; observations of EOC processes and interviews with staff and 999 callers. All interviews in the main trial site and observational substudy will be digitally recorded and transcribed verbatim. Once the transcripts have been checked for accuracy, recordings will be deleted. Transcripts will be analysed thematically using the Framework Method.^33^ Data will be analysed concurrent to its collection where possible and will inform the topic guides for interviews.

#### Health economic analysis

A health economic analysis will be undertaken from NHS and societal perspectives to inform the design of a full economic evaluation within the future RCT. The primary purpose of the health economic analysis is to assess the feasibility of gathering data on the resource implications, costs and effects of the dispatch decisions under standard care and when adjusted for the live streaming intervention.

### Data monitoring

A data monitoring committee was not required as this study is a feasibility study and there is no interim analysis.

### Harms

The study teams and participating staff at EOCs will inform the Chief Investigator and/or Sponsor as soon as they are aware of a possible serious breach that could impact the safety, or physical or mental integrity of subjects/participants in the study, so that the Sponsor can fulfil its regulatory and oversight requirements. Other deviations from the protocol will be recorded and dealt with appropriately.

### Auditing

The research team at each study site will be responsible for ensuring the accuracy and quality of data collection and data entry. Several data audit checks will be conducted by the RPs prior to transfer of the study database to the university and analysis to ensure accurate data recording.

### Committees

#### Steering Committee (SC)

The SC will monitor progress against milestones, spend against budget, provide advice where necessary, promote the project, facilitate communication between organisations with stakeholders and help maximise dissemination and impact of findings. Membership will be independently appointed and NIHR-approved.

#### Project Advisory group (PAG)

The project will be supported by a PAG, chaired by the Patient and Public Involvement and Engagement (PPIE) lead who will also chair a separate but inter-connected PPIE group. The PAG will meet up to four times over the course of the study to coincide with key timepoints in the project and provide a forum for input and support regarding the data collection, analysis and production of outputs and dissemination.

#### PPIE group

The PPIE group will be formed of up to five lay representatives and be chaired by a lay public PPIE lead. We will ask for their assistance with developing patient-facing materials and input into strategic and management decisions. We will involve the PPIE group during data analysis, through sense-checking emerging results and considering how to interpret findings. Finally, we will work with the PPIE group to determine the most effective ways of dissemination, using a variety of approaches from journal publications and conference presentations, through to use of social media sites and a short film.

## Ethics and dissemination

### Research ethics approval

Favourable ethical opinion was granted by London—Camden & King’s Cross Research Ethics Committee (reference 21/LO/0912).

### Protocol amendments

Amendments to the protocol will be updated on the ISRCTN registry, uploaded to the NIHR project webpage and shared with all study sites and key members of the research team.

### Confidentiality

All trial data will be anonymised prior to transfer to the University for analysis. Qualitative data (interviews, observational field notes) will be anonymised as soon as possible after collection and only anonymised data will be analysed. Any quotes used to illustrate findings will be unidentifiable to the source.

### Access to data

Direct access will be granted to authorised representatives from the Sponsor, host institution and regulatory authorities to permit trial-related monitoring, audits and inspections, in line with participant consent. Access to the final dataset will be restricted to members of the research team. The data will be kept securely and in a pseudo-anonymised format to protect personal sensitive data from being associated with any individual or participant.

### Dissemination

The main output of this feasibility RCT will be the knowledge gained regarding the acceptability and feasibility of using live streaming in practice and the learning about data collection and research processes to inform the development of a larger multicentre RCT. Findings will be shared with key stakeholders (eg, policy makers, commissioners, providers, emergency response staff, patients and public, academic audiences) by a wide variety of means, including journal publications, conference presentations, summary reports, media releases, the project webpage (www.surrey.ac.uk/seeitstudy), social media and a short film. Authorship of outputs will be decided using the International Committee of Medical Journal Editors criteria^34^ and has been agreed with all members of the research team. The full dissemination policy can be found in the published protocol on the NIHR webpage.^22^

## Supplementary Material

Reviewer comments

Author's manuscript
