# Supplementary material for: The SEE-IT Trial: emergency medical services Streaming Enabled Evaluation In Trauma: study protocol for an interventional feasibility randomised controlled trial
Source: BMJ Open. 2023 Apr 24;13(4):e072877. doi: 10.1136/bmjopen-2023-072877 (PMC10151834; doi:10.1136/bmjopen-2023-072877)
Supplement: Author's manuscript [file bmjopen-2023-072877.draft_revisions.pdf]

BMJ Open

BMJ Open is committed to open peer review. As part of this commitment we make the peer review history of every article we publish publicly available.

When an article is published we post the peer reviewers' comments and the authors' responses online. We also post the versions of the paper that were used during peer review. These are the versions that the peer review comments apply to.

The versions of the paper that follow are the versions that were submitted during the peer review process. They are not the versions of record or the final published versions. They should not be cited or distributed as the published version of this manuscript.

BMJ Open is an open access journal and the full, final, typeset and author-corrected version of record of the manuscript is available on our site with no access controls, subscription charges or pay-per-view fees (http://bmjopen.bmj.com).

If you have any questions on BMJ Open's open peer review process please email
info.bmjopen@bmj.com

BMJ Open

The SEE-IT Trial: Emergency Medical Services Streaming Enabled Evaluation In Trauma: study protocol for an interventional feasibility randomised controlled trial

Journal:	*BMJ Open*
Manuscript ID	bmjopen-2023-072877
Article Type:	Protocol
Date Submitted by the Author:	16-Feb-2023

Complete List of Authors:	Ollis, Lucie; University of Surrey School of Health Sciences, School of Health Sciences<br>Skene, Simon; University of Surrey, Surrey Clinical Trials Unit<br>Williams, Julia; South East Coast Ambulance Service NHS Foundation Trust; University of Hertfordshire School of Health and Social Work<br>Lyon, Richard; University of Surrey School of Health Sciences; Kent, Surrey & Sussex Air Ambulance<br>Taylor, Cath; University of Surrey School of Health Sciences<br>The SEE-IT Trial Group, The SEE-IT Trial Group; University of Surrey; South East Coast Ambulance Service NHS Foundation Trust

Keywords:	ACCIDENT & EMERGENCY MEDICINE, Telemedicine < BIOTECHNOLOGY & BIOINFORMATICS, Information technology < BIOTECHNOLOGY & BIOINFORMATICS, Feasibility Studies, PRIMARY CARE, TRAUMA MANAGEMENT

SCHOLARONE™
Manuscripts

The SEE-IT Trial: Emergency Medical Services **S**treaming **E**nabled **E**valuation **I**n **T**rauma: study protocol for an interventional feasibility randomised controlled trial

Lucie Ollis, Simon Skene, Julia Williams, Richard Lyon, and Cath Taylor on behalf of the SEE-IT Trial Group

SEE-IT Trial Group to include:

Kate Bennett-Eastley, Mark Cropley, Heather Gage, Janet Holah, Jill Maben, Carin Magnusson, Craig Mortimer, and Scott Munro.

Corresponding author: Professor Cath Taylor, School of Health Sciences, Kate Granger Building, University of Surrey, Guildford, Surrey, GU2 7XH, cath.taylor@surrey.ac.uk

For peer review only - http://bmjopen.bmj.com/site/about/guidelines.xhtml

ABSTRACT

Introduction

Accurate and timely dispatch of emergency medical resources (EMS) is vital due to limited resources and patients' risk of mortality and morbidity increasing with time. Currently, most UK Emergency Operations Centres (EOCs) rely on audio calls and accurate descriptions of the incident and patients' injuries from lay 999 callers. If dispatchers in the EOCs could see the scene via live video streaming from the caller's smartphone, this may enhance their decision making and enable quicker and more accurate dispatch of EMS. The main aim of this feasibility randomised controlled trial (RCT) is to assess the feasibility of conducting a definitive RCT to assess the clinical and cost effectiveness of using live streaming to improve targeting of EMS.

Methods and analysis

The SEE-IT Trial is a feasibility RCT with a nested process evaluation. The study also has two observational sub-studies: (i) in an EOC who routinely use live streaming to assess the acceptability and feasibility of live streaming in a diverse inner-city population and (ii) in an EOC that does not currently use live streaming to act as a comparator site regarding the psychological wellbeing of EOC staff using vs not using live streaming.

Ethics and dissemination

Favourable ethical opinion was granted by the Health Research Authority on 23/03/2022 (ref: 21/LO/0912), including NHS Confidentiality Advisory Group (CAG) approval received on 22/03/2022 (ref: 22/CAG/0003). This manuscript refers to version 0.8 of the protocol (07/11/2022). The trial is registered with the ISRCTN (ISRCTN11449333). The first participant was recruited on 18 June 2022.

The main output of this feasibility trial will be the knowledge gained to help inform the development of a large multi-centre RCT to evaluate the clinical and cost effectiveness of the use of live streaming to aid EMS dispatch for trauma incidents.

For peer review only

Strengths and limitations of this study

- This is the first RCT of the use of live streaming for major trauma and includes a comprehensive process evaluation to understand acceptability and impact of using live streaming from the perspective of 999 callers and emergency operations staff.
- The mixed methods approach to evaluation of feasibility and acceptability is a key strength, using a combination of routinely collected data, observational data, interviews, and survey methods.
- The design (testing for a week each month over 6 months) builds consideration of seasonal variation in trauma into the design, whilst enabling time to adapt and improve the protocol as the SEE-IT Trial progresses.
- The Ambulance Trust where live streaming will be tested uses NHS Pathways (as used in half of Ambulance Trusts in UK); the SEE-IT Trial will give us insight into how live streaming would work with this dispatch system but may not extrapolate fully to areas that use other systems for triage/decision support.

For peer review only

INTRODUCTION

Background and Rationale

Emergency medical services (EMS) provide emergency medical care at the scene of incidents before patients are conveyed to hospital (e.g., blood transfusions and emergency anaesthesia). To save lives and prevent long term disability, timely and accurate dispatch of pre-hospital resources is critical (1). Evidence suggests trauma patients should receive definitive care within one hour of the incident (The 'Golden Hour'), otherwise the risk of morbidity and mortality increases significantly (2). Where severe injury is suspected by EMS (e.g., major trauma), specialist paramedics in critical care and/or Helicopter Emergency Medical Services (HEMS) may be dispatched to the scene to care for the patient and/or convey the patient to hospital.

When a 999 call is made in the UK, the dispatchers in the Emergency Operations Centre (EOC) need to decide which resources to send to the scene by gathering information about what has happened and the clinical state of the patient(s). The dispatchers rely on 999 callers and other bystanders to accurately relay essential information (3). However, lay public 999 callers can sometimes provide inaccurate information due to language barriers, subjectivity, the emotional impact of being at the scene and limited medical training or knowledge (3,4). Dispatch response is often recognised as a weak link in the EMS chain (5), often involving either under-resourcing or over-resourcing (6–9). Previous studies have shown that up to 50% of HEMS (air ambulance) dispatch and 25% of road ambulance dispatch for suspected trauma patients are not appropriate (6–9).

It is vital that EMS are being dispatched to incidents where they are needed due to finite and often limited resources. If dispatchers were able to see the patient and scene via live video streaming through a 999 callers' phone camera, it could be easier to make more accurate and timely decisions about dispatch of EMS (4). There is evidence that video-assisted CPR has the potential to improve patient outcomes (10–16), but there is a lack of research evidence to support the use of live video streaming for EMS dispatch for trauma.

NHS policies and the Department of Health and Social Care actively encourage the use of innovative technologies to improve patient and health care outcomes (17). The benefits of using video in other healthcare settings is growing, e.g. for remote healthcare consultations (18–21), but evidence is sparse in relation to use in pre-hospital settings, despite being used routinely in some ambulance services. There is some limited evidence of the benefits and

For peer review only - http://bmjopen.bmj.com/site/about/guidelines.xhtml

acceptability of live streaming to aid HEMS dispatch (4), but its impact on clinical or economic outcomes has not yet been evaluated. Also, the psychological impact of dispatchers viewing the scene and impact on 999 callers has not been investigated for exposure to trauma.

Objectives and research questions

Main research question: Is it feasible to conduct a future randomised controlled trial (RCT) to assess the clinical and cost effectiveness of using GoodSAM live video streaming to improve targeting of emergency medical resources? The overall aim of this research is to assess the feasibility of implementing and evaluating GoodSAM live streaming in a definitive RCT.

The objectives are:

(i) To obtain data required to inform the design of a subsequent RCT (e.g., event rate, screening rate, effect size/precision for outcomes, health economic data).

(ii) To test trial processes including randomisation and data collection methods.

(iii) To conduct a nested process evaluation to test the acceptability and feasibility of using GoodSAM live streaming from provider and public perspectives (e.g., training, video feasibility, video acceptability, psychological harm to 999 callers and/or dispatch staff).

Further research questions relating to these objectives can be found in the study protocol published on the National Institute for Health and Care Research (NIHR) study webpage (22).

METHODS AND ANALYSIS

Trial design and study setting

The SEE-IT Trial is a feasibility RCT with a nested process evaluation in an EOC in South East England. This study includes two observational sub-studies (i) in an EOC who routinely use live streaming to assess acceptability and feasibility of use of live streaming in a diverse inner-city population and (ii) in an EOC that does not currently use live streaming to act as a comparator site regarding the psychological wellbeing of EOC staff using vs not using live streaming. In addition, eleven NHS hospital trusts covered by the main trial site will be involved in identifying and consenting patients for access to their medical records. A full list of the study sites can be found via the protocol on the NIHR study webpage (22).

For peer review only - http://bmjopen.bmj.com/site/about/guidelines.xhtml

The study was planned to run for 18 months from 1st October 2021 to 31st March 2023. Pressures on the NHS and ambulance services meant the start of the trial period was delayed 4 months to June 2022. Therefore, the overall study end date is now 31st July 2023. There were nine amendments made to the protocol during the feasibility study, outlined in the protocol on the NIHR study webpage (22).

Eligibility criteria

This study includes three types of participants; trauma patients, lay public 999 callers and EOC staff. See tables 1 and 2 for inclusion and exclusion criteria respectively.

Table 1: Participant inclusion criteria

Participant type	Main feasibility trial	Inner-city observational sub-study	Staff wellbeing sub-study
**Trauma patients**	All trauma patients during the six trial observation weeks who are the subject of 999 calls involving major trauma, judged by the HEMS dispatcher and/or Critical Care Paramedics (CCPs) as likely to require enhanced dispatch.	All trauma patients during observed shifts that involve trauma and are screened by HEMS dispatchers or critical care advanced paramedic practitioner dispatchers (APPs) who attempt to use GoodSAM during the call.	N/A
**Lay public 999 callers**	999 callers during the six trial weeks where the incident involves major trauma (defined as per above).	All 999 callers during observed shifts that involve trauma and are screened by HEMS dispatchers or critical care APPs who attempt to use GoodSAM during the call.	N/A
**EOC staff**	All CCPs, HEMS dispatchers, and research paramedics.	All HEMS dispatchers and critical care APPs.	All CCPs and HEMS dispatchers.

Note: CCPs in the main feasibility trial site and APPs in the inner-city observational sub study are the ways different Ambulance Trusts describe specialist paramedics in critical care.

For peer review only - http://bmjopen.bmj.com/site/about/guidelines.xhtml

Table 2: Participant exclusion criteria

Participant type	Main feasibility trial	Inner-city observational sub-study	Staff wellbeing sub-study
**Trauma patients**	Any emergencies of a suspected medical origin (e.g., heart attack or stroke).	Any emergencies of a suspected medical origin (e.g., heart attack or stroke).	N/A
**Lay public 999 callers**	Calls will be excluded where: (i) 999 caller is not at the scene; (ii) 999 call originates from a landline; (iii) 999 call originates from another emergency service e.g. police or fire; (iv) 999 calls where resource (excluding community first responder, CFR) will arrive on scene before live streaming could be activated; (v) 999 call ended before transfer for activation of live streaming; (vi) 999 calls where another incident takes priority; and (vii) calls where clinical acuity is found to be lower than the threshold for the study (not major trauma).<br>All callers identified by the dispatchers as a child caller (under 16 years old) and those who select they are under 16 on the 999-caller survey will be excluded.	All callers identified by the dispatchers as a child caller (under 16 years old) and those who select they are under 16 on the 999-caller survey will be excluded.	N/A
**EOC staff**	EOC staff not mentioned in the inclusion criteria	EOC staff not mentioned in the inclusion criteria	EOC staff not mentioned in the inclusion criteria

Intervention and randomisation

999 calls in the main trial site during the six observation weeks (up to 42 days; 84 shifts) will be randomised 1:1 to working shift intervention or standard care. Control shifts will follow standard care ambulance dispatch protocol with a 999 caller using a telephone (voice only) and the call taker using NHS Pathways ambulance dispatch tool.

For peer review only - http://bmjopen.bmj.com/site/about/guidelines.xhtml

In the intervention arm, a live streaming technology called GoodSAM Instant-on-Scene™ (23) will be tested. The technology allows a 999 caller to stream live video footage from the scene of an incident to the EOC. 999 calls allocated to intervention will initially follow the standard NHS Pathways dispatch protocol until ambulance dispatch prioritisation has been determined. EMS will be dispatched as normal, without delay. The call handler in the EOC will then transfer the caller to the HEMS dispatcher or Critical Care Paramedic (CCP) and live streaming will be activated. EMS resource allocation may be adjusted following this.

If at any point the dispatcher feels they do not want to view the scene (e.g., due to psychological impact of viewing the scene) or it is not safe to continue, the 999 caller will be thanked for their help and the call will be ended. The dispatchers will be provided with training and guides outlining the trial processes to ensure adherence to intervention and study protocols.

Outcomes

Primary outcomes

The primary outcome is the decision regarding the feasibility of undertaking a definitive RCT based primarily on meeting the specified progression criteria (see table 3).

Table 3: Progression criteria from feasibility RCT to future RCT

GREEN; proceed to definitive study - GO	AMBER; consider protocol amendments to improve criteria	RED; do not proceed to main trial - STOP
≥70% of callers with smartphones agreeing and able to activate live streaming	≥50% of callers with smartphones agreeing and able to activate live streaming	<50% of callers with smartphones agreeing and able to activate live streaming
≥50% of requests to activate live streaming resulting in footage being viewed	≥30% but <50% of requests to activate live streaming resulting in footage being viewed	<30% of requests to activate live streaming resulting in footage being viewed
Air Ambulance (HEMS) stand-down rate reducing by ≥10% and/or change in dispatch decision as a result of live streamed footage confirmed as being appropriate in ≥10% cases	Air Ambulance stand-down rate reducing by ≥5% and/or change in dispatch decision as a result of live streamed footage confirmed as being appropriate in ≥5% cases	No change in Air Ambulance stand-down rate and/or change in dispatch decision as a result of live streamed footage

For peer review only - http://bmjopen.bmj.com/site/about/guidelines.xhtml

Rates of psychological harm (based on the survey measures) not significantly greater in 999 callers using live streaming compared to those not; and no significant difference in change to psychological harm over time in staff (CCPs, HEMS dispatchers, Research Paramedics) compared to change in staff in a comparison EOC not using live streaming/streamed from scene footage	-	Evidence of significantly greater harm in either 999 callers or EOC staff using live streaming compared to EOC not using live streaming.

In addition to the quantitative progression criteria described in table 3, the research team will consider qualitative data collected as part of this study (e.g., interviews, observations, and free text questions in surveys) when reviewing progression to a definitive RCT.

Secondary outcomes

Speed of appropriate EMS dispatch: speed will be measured using time-stamped data from start of 999 calls to appropriate deployment with judgement of 'appropriateness' to be based on algorithms developed through expert consensus (see below) and using medical records data up to 3 months post-incident where consent is provided; Stand-down rate (de-escalation); missed jobs (e.g. not prioritised for HEMS/CCP despatch, either due to lack of resource or inappropriate prioritisation); requests for further ambulance resources from scene. Psychological harm will be assessed using two validated scales (24, 25) pre and post-trial in 999 callers from both arms and staff viewing the footage (including staff in comparison EOC).

The appropriateness of dispatch will be determined based on algorithms developed through expert consensus before the trial starts. Appropriateness is defined as the need for intervention and/or transport by the response vehicle/staff dispatched to scene (e.g. for HEMS dispatch that a critical care intervention and/or transportation by helicopter was required (9)). A binary outcome decision tree will be developed with the reasons for appropriate/inappropriate dispatch categorised and agreed (inappropriate dispatch will be further categorised as being 'over' or 'under' resourced).

Participant timeline

There will be six trial weeks, one week a month from June to November 2022. Each trial week will include up to 14 randomised shifts (day and night). EOC staff in the main trial site and staff wellbeing sub study will be invited to participate in the staff survey pre- and post-

For peer review only - http://bmjopen.bmj.com/site/about/guidelines.xhtml

trial period. Staff in the main trial site will be invited to partake in interviews through the survey. 999 callers in the main trial site will be sent a text message after the eligible incident inviting them to participate in a survey 6-8 weeks post incident and a subsequent interview. Patients will be approached for consent for their medical records to be accessed up to 3 months after the incident has occurred.

In the observational sub-study, EOC staff will be observed using GoodSAM live streaming over a 3-month period and invited to partake in interviews. 999 callers will be invited to partake in the survey within 1 week of the incident they called 999 for and subsequently invited to partake in an interview about their experiences of using GoodSAM live streaming.

Sample size

We estimate there will be approximately 250 trauma incidents (including 300 patients) over the six observation weeks (125 allocated to intervention), which will allow for an estimate of true event rate within precision of +/-0.75 events per day; and allow estimation of speed to appropriate response with a standard error of <5%. These sample size estimations are based on convenience sampling over the six defined trial observation weeks. We estimate there will be approximately 350 lay public 999 callers (250 from the main trial site and 100 from the observational sub-study) and up to 202 EOC staff (86 from the main trial site, 30 from the observational sub-study and 86 from the third EOC).

Recruitment: Identification and consent

Trauma patients

Trauma patients will be identified by Research Paramedics (RPs), using the eligibility criteria. RPs will observe all trial shifts. For eligible calls in the main trial site, the Computer Aided Dispatch (CAD) number(s) relating to the incident will be recorded by the RP as the identifier to share with the conveying hospital to approach for consent. Informed consent will be sought from either the patient, their consultee or the patient's parent or guardian (either in the hospital before discharge or via telephone, email, or post) in all hospital trusts included in the trial (trauma units and major trauma centres covered by the EOC). RPs may consent patients in person with assistance from the hospital to identify the patient(s). Approval has been granted by the Confidentiality Advisory Group (Section 251 application) to access medical records for casualties who die before consent can be sought (and where there is not a

For peer review only - http://bmjopen.bmj.com/site/about/guidelines.xhtml

power of attorney in place for that individual). National data opt-out status will be checked for all deceased patients.

999 callers

In the main feasibility site, the RP will determine eligibility of the 999 callers and prompt the dispatchers to send a text message via the CAD, inviting the 999 caller to participate in a survey about their experience of calling 999 for the incident. The text message will include a link to the PIS, consent form and contact details for the research team. A reminder text message will be sent to 999 callers within one week of the incident. Those who participate in the survey will be invited to partake in an interview and selected to represent a range in demographics (age, ethnicity, gender), type of incident they witnessed and evidence or not of psychological harm from survey responses.

In the inner-city observational sub-study, at the end of the call (once resources have been dispatched) 999 callers will be asked for permission to store their name and number to share with the researcher to be invited to participate in the study. Those who consent will initially be contacted by phone so the researcher can explain the study and ask their permission to send them further details about the study via text message or email. The researcher will then send a text message or email with a link to the PIS and consent form, which once completed will re-direct to the survey. Reminder texts/emails will be sent up to one week after the incident. Only those who participate in the survey will be invited to partake in an interview.

EOC staff

In the main trial site, the use of live streaming has been approved at organisation level and thereby staff are not individually consented to use live streaming but instead will be given the opportunity to opt out of trial shifts. They will otherwise be required to follow organisational protocols if they are working on a trial shift. In the observational sub-study, HEMS dispatchers and critical care APPs will consent on an individual basis to being observed using live streaming. The researcher will contact staff to invite them to participate in an interview about the use of live streaming in their organisation. The local PI at both EOCs participating in the staff survey will share the staff survey link with all eligible staff pre-trial. All staff who enter their email address to complete the post-trial survey will be emailed a link to the survey post-trial. Those who participate in the staff survey at the main trial site will be invited to partake in an interview about their views and experiences of live streaming; selected to include different types of staff e.g., age, gender, and experience.

For peer review only - http://bmjopen.bmj.com/site/about/guidelines.xhtml

Assignment of intervention

Working shifts are randomised 1:1 to intervention (GoodSAM) or standard ambulance care using a minimisation algorithm to ensure balance between weekday/weekend (Mon-Thurs vs Fri-Sun) and daytime/night-time shifts (0600-1800 vs 1800-0600).

A small number of shifts in the final observation week will be used to test the feasibility of individual call randomisation using a pre-prepared randomisation list. All randomisations will be undertaken by a statistician at Surrey CTU to ensure the allocation is independent of the research team.

Data collection methods

Observations of EOC processes

An experienced researcher will observe EOC processes, decision making processes, fidelity to the protocol and impact of contextual variability (e.g., time of day, weather) during the trial weeks in the main trial site (3-6 hours for 3 days in each observation week). In the observational sub-study, a second experienced researcher will observe up to 24 shifts over a 3-month period. Observational data will be recorded using fieldnotes capturing both descriptive and conceptual observations/interpretations.

The observational and interview data collection and analysis will be underpinned by theories informing the proposed mechanisms of effect e.g. Situational Awareness, SA (26); Decision Making, DM (27); the implementation of a new technology, TAM (28–30); and Consolidated Framework for Implementation Research, CFIR (31). Events will be observed in real time and the use of live streaming will be understood in the context of how the use of visual technology impacts on staff assessment of situations and action taken (SA and DM), as well as the factors influencing how people relate to the introduction of a new technology (TAM). Equally, in situations where live streaming is not used, theories of SA and DC will inform observations in context.

Decision making data and clinical data from trauma patients

During the trial weeks, the RPs will collect data live during incidents including the CAD number; information about decision making; the patients' name, age, and sex (if available); details of the trauma incident (e.g., single/multiple casualties; general nature of incident e.g., road traffic collision/assault); dispatch decisions; and the use of live streaming e.g., quality of

For peer review only - http://bmjopen.bmj.com/site/about/guidelines.xhtml

sound/picture. This information will be recorded on a piloted, study-specific proforma completed at the time of the incident. Further details about EMS dispatch (including speed to appropriate dispatch) and time stamped data for each incident will be accessed by RPs after the incident.

Following consent to access medical records (process described in recruitment section), the RPs will extract relevant information from patients' medical records about the injuries sustained and the treatments received (up to 3 months post-incident). Using the expert panel algorithms, the RPs will rate the appropriateness of dispatch. A random 10% sample of incidents from intervention and control calls will be independently reviewed by the expert panel using the same data, to assess reliability.

999 callers

999 callers in the main trial site will be invited to complete surveys via Qualtrics online survey software (32) 6-8 weeks post incident to assess psychological harm and semi-structured interviews regarding experiences and acceptability of the use of live streaming. The questionnaire will include demographic questions, The Impact of Events Scale - Revised (24), The General Health Questionnaire-12 item version (25) and questions about acceptability and experience of live streaming.

In the inner-city observational sub-study, 999 callers will be invited to complete surveys via Qualtrics (32) up to one week after the incident. The questionnaire will include a demographic questionnaire and questions about their experiences of using live streaming during their recent 999 call e.g., ease of use, usefulness. Those who take part in the questionnaire will be invited to participate in an interview regarding experiences and acceptability of using live streaming.

999 callers at both sites who participate in the survey will be offered the choice of a £10 shopping voucher or £10 donation to the local air ambulance charity. A second voucher or donation will be offered to those who complete an interview.

EOC staff survey and interviews

Before the trial starts, EOC staff at the main trial site and staff wellbeing sub-study will be emailed by the local PI including the PIS, consent form and a link to the online survey via Qualtrics (32). The survey will include a demographic questionnaire, The Impact of Events Scale - Revised (24), The General Health Questionnaire-12 item version (25) and questions

For peer review only - http://bmjopen.bmj.com/site/about/guidelines.xhtml

about live streaming. Those who complete both pre- and post-trial questionnaires will be entered into a prize draw for one of five £50 retail vouchers (five available at each site). In the main trial site, approximately 12-18 staff will be invited to participate in an interview and will be paid overtime rates for their participation. Staff in the observational sub-study who take part in an interview will be compensated for their time with a £45 Amazon voucher. The different methods for reimbursing time have been requested by the respective organisations.

Data management

A detailed data management plan has been produced outlining the processes for completion, transfer, and storage of study data in line with the Ambulance Trust and University policies, the requirements of the Sponsor and GDPR. The data management plan will be shared with all those responsible for data collection on the trial and will form the basis of a Data Protection Impact Assessment (DPIA) and data processing agreements between the parties.

Statistical methods and data analysis

Quantitative analysis

A full Statistical Analysis Plan (SAP) will be reviewed by key members of the research team and approved by the Steering Committee before analysis is undertaken.

As this is a feasibility study, statistical analysis will focus on providing estimates and confidence intervals of key rates such as the number of eligible calls per day, uptake of video intervention and timing and appropriateness of dispatch to inform a subsequent RCT. Outcome measure will be summarised by arm and estimates reported with confidence intervals to inform future sample size calculations.

The SAP will outline any expected exploratory analysis of outcomes that may be useful to inform a subsequent RCT including any signal of potential efficacy, but it is noted that the study is not powered for this and so any interpretation would be limited to the direction and magnitude of any effect. Missing data (including outcome data) will be summarised, but all observed data will be included in any analysis according to the randomised allocation following the intention-to-treat principle.

Qualitative analysis

For peer review only - http://bmjopen.bmj.com/site/about/guidelines.xhtml

For peer review only

The process evaluation will include analysis of live streaming usage data; observations of EOC processes; and interviews with staff and 999 callers. All interviews in the main trial site and observational sub-study will be digitally recorded and transcribed verbatim. Once the transcripts have been checked for accuracy, recordings will be deleted. Transcripts will be analysed thematically using the Framework Method (33). Data will be analysed concurrent to its collection where possible and will inform the topic guides for interviews.

Health economic analysis

A health economic analysis will be undertaken from NHS and societal perspectives to inform the design of a full economic evaluation within the future RCT. The primary purpose of the health economic analysis is to assess the feasibility of gathering data on the resource implications, costs, and effects of the dispatch decisions under standard care and when adjusted for the live streaming intervention.

Data Monitoring

A data monitoring committee was not required as this study is a feasibility study and there is no interim analysis.

Harms

The study teams and participating staff at EOCs will inform the Chief Investigator and/or Sponsor as soon as they are aware of a possible serious breach that could impact the safety, or physical or mental integrity of subjects/participants in the study, so that the Sponsor can fulfil its regulatory and oversight requirements. Other deviations from the protocol will be recorded and dealt with appropriately.

Auditing

The research team at each study site will be responsible for ensuring the accuracy and quality of data collection and data entry. Several data audit checks will be conducted by the RPs prior to transfer of the study database to the university and analysis to ensure accurate data recording.

For peer review only - http://bmjopen.bmj.com/site/about/guidelines.xhtml

Committees

Steering Committee (SC)

The SC will monitor progress against milestones, spend against budget, provide advice where necessary, promote the project, facilitate communication between organisations with stakeholders, and help maximise dissemination and impact of findings. Membership will be independently appointed and NIHR-approved.

Project Advisory Group (PAG)

The project will be supported by a PAG, chaired by the PPIE lead who will also chair a separate but inter-connected PPIE Group. The PAG will meet up to 4 times over the course of the study to coincide with key timepoints in the project and provide a forum for input and support regarding the data collection, analysis, and production of outputs and dissemination.

Patient and Public Involvement and Engagement Group (PPIE)

The PPIE group will be formed of up to five lay representatives and be chaired by a lay public PPIE lead. We will ask for their assistance with developing patient-facing materials and input into strategic and management decisions. We will involve the PPIE group during data analysis, through sense-checking emerging results and considering how to interpret findings. Finally, we will work with the PPIE group to determine the most effective ways of dissemination, using a variety of approaches from journal publications and conference presentations, through to use of social media sites and a short film.

ETHICS AND DISSEMINATION

Research Ethics Approval

Favourable ethical opinion was granted by London – Camden & King's Cross Research Ethics Committee (reference 21/LO/0912).

Protocol amendments

Amendments to the protocol will be updated on the ISRCTN registry, uploaded to the NIHR project webpage, and shared with all study sites and key members of the research team.

Confidentiality

All trial data will be anonymised prior to transfer to the University for analysis. Qualitative data (interviews, observational field notes) will be anonymised as soon as possible after

For peer review only - http://bmjopen.bmj.com/site/about/guidelines.xhtml

collection and only anonymised data will be analysed. Any quotes used to illustrate findings will be unidentifiable to the source.

Declaration of Interests

None declared.

Access to Data

Direct access will be granted to authorised representatives from the Sponsor, host institution and regulatory authorities to permit trial-related monitoring, audits, and inspections, in line with participant consent. Access to the final dataset will be restricted to members of the research team. The data will be kept securely and in a pseudo-anonymised format to protect personal sensitive data from being associated with any individual or participant.

Dissemination

The main output of this feasibility RCT will be the knowledge gained regarding the acceptability and feasibility of using live streaming in practice and the learning about data collection and research processes to inform the development of a larger multi-centre RCT. Findings will be shared with key stakeholders (e.g., policy makers, commissioners, providers, emergency response staff, patients and public, academic audiences) by a wide variety of means, including journal publications, conference presentations, summary reports, media releases, the project webpage (www.surrey.ac.uk/seeitstudy), social media and a short film. Authorship of outputs will be decided using the International Committee of Medical Journal Editors criteria (ICMJE (34)) and has been agreed with all members of the research team. The full dissemination policy can be found in the published protocol on the NIHR webpage (22).

ADMINISTRATIVE INFORMATION

Author contributions: The original protocol was drafted by CT and SS, with significant contributions from JW and RL, and based on the original grant proposal contributed to by all co-applicants. The manuscript was drafted by LO, and critically reviewed by all other authors named in the SEE-IT Trial Group. All authors approved the final version of the protocol.

Trial sponsor: The trial Sponsor is the University of Surrey Research Integrity and Governance Office (reference SPON 2021 22 FHMS, rigo@surrey.ac.uk, 01483 683490) with trial management activities conducted by the University of Surrey Clinical Trials Unit.

For peer review only - http://bmjopen.bmj.com/site/about/guidelines.xhtml

Funding statement: This work was funded by the National Institute of Health Research (NIHR, award number 130811, https://fundingawards.nihr.ac.uk/award/NIHR130811). The trial sponsor and funding body contributed to the design of this study but will not have any role during its execution, analysis, interpretation if the data, writing of the report or the decision to submit the report for publication.

Competing interests: None declared.

Acknowledgments: We would like to thank all have supported this study within Air Ambulance Charity Kent, Surrey, Sussex; South East Coast Ambulance Service NHS Foundation Trust; London Ambulance Service; East of England Ambulance Service; and the major trauma centres and units in the region. In addition, we would like to thank our PPIE Group; Project Advisory Group; Steering Committee and Expert Panel members for their valuable contributions. Finally, we would like to thank Oliver Fitzgerald, Fon Yan Li, Andrew Fooks, Caroline Cripps, and Anthony Coe for contributing to data collection; Rachael Cooke for the project administrative support; and Matthew Glover for contributing to the health economic methods.

For peer review only

REFERENCES

1. Duke M, Tatum D, Sexton K et al. When Minutes Fly by: What is the True "Golden Hour" for Air Care? Am Surg. 2018 Jun;84(6):862–7. doi: 10.1177/000313481808400633

2. Pham H, Puckett Y, Dissanaike S. Faster on-scene times associated with decreased mortality in Helicopter Emergency Medical Services (HEMS) transported trauma patients. Trauma Surg Acute Care Open. 2017 Oct 1;2(1):e000122. doi: 10.1136/tsaco-2017-000122

3. Carroll LN, Calhoun RE, Subido CC et al. Serving Limited English Proficient Callers: A Survey of 9-1-1 Police Telecommunicators. Prehospital Disaster Med. 2013 Jun;28(3):286–91. doi: 10.1017/S1049023X13000265

4. ter Avest E, Lambert E, de Coverly R et al. Live video footage from scene to aid helicopter emergency medical service dispatch: a feasibility study. Scand J Trauma Resusc Emerg Med. 2019 Dec;27(1):55. doi: 10.1186/s13049-019-0632-4

5. Schellhaaß A, Popp E. Air rescue: current significance and practical issues. Anaesthesist. 2014 Dec 1;63(12):971–80; quiz 981–2. doi: 10.1007/s00101-014-2356-1

6. Eaton G, Raitt J, Brown S et al. How appropriate are the data produced by NHS Pathways for identifying suitable cases for involvement of enhanced care teams? Br Paramed J. 2017 Dec 1;2(3):16–23. doi: 10.29045/14784726.2017.2.3.16

7. Palazzo FF, Warner OJ, Harron M et al. Misuse of the London ambulance service: How much and why? Emerg Med J. 1998 Nov 1;15(6):368–70. doi: 10.1136/emj.15.6.368

8. Wilmer I, Chalk G, Davies GE et al. Air ambulance tasking: mechanism of injury, telephone interrogation or ambulance crew assessment?: Table 1. Emerg Med J. 2015 Oct;32(10):813–6. doi: 10.1136/emermed-2013-203204

9. Munro S, Joy M, de Coverly R, et al. A novel method of non-clinical dispatch is associated with a higher rate of critical Helicopter Emergency Medical Service intervention. Scand J Trauma Resusc Emerg Med. 2018 Sep 25;26(1):84. doi: 10.1186/s13049-018-0551-9

10. Kim GW, Lee DK, Kang BR et al. A multidisciplinary approach for improving the outcome of out-of-hospital cardiac arrest in South Korea. Eur J Emerg Med Off J Eur Soc Emerg Med. 2020 Feb;27(1):46–53. doi: 10.1097/MEJ.0000000000000612

11. Johnsen E, Bolle SR. TO SEE OR NOT TO SEE—Better dispatcher-assisted CPR with video-calls? A qualitative study based on simulated trials. Resuscitation. 2008 Sep;78(3):320–6. doi: 10.1016/j.resuscitation.2008.04.024

12. Huang HK, Chen HH, Chen YL et al. A Novel Assessment Using a Panoramic Video Camera of Resuscitation Quality in Patients following Out-of-Hospital Cardiac Arrest. Prehospital Emerg Care Off J Natl Assoc EMS Physicians Natl Assoc State EMS Dir. 2022 Jan 25;1–4. doi: 10.1080/10903127.2021.2015025

13. Lee HS, You K, Jeon JP et al. The effect of video-instructed versus audio-instructed dispatcher-assisted cardiopulmonary resuscitation on patient outcomes following out of

For peer review only - http://bmjopen.bmj.com/site/about/guidelines.xhtml

hospital cardiac arrest in Seoul. Sci Rep. 2021 Jul 30;11(1):15555. doi: 10.1038/s41598-021-95077-5

14. Linderoth G, Rosenkrantz O, Lippert F et al. Live video from bystanders' smartphones to improve cardiopulmonary resuscitation. Resuscitation. 2021 Nov;168:35–43. doi: 10.1016/j.resuscitation.2021.08.048

15. Linderoth G, Møller TP, Folke F, Lippert FK, Østergaard D. Medical dispatchers' perception of visual information in real out-of-hospital cardiac arrest: a qualitative interview study. Scand J Trauma Resusc Emerg Med. 2019 Jan 25;27(1):8. doi: 10.1186/s13049-018-0584-0

16. Lin YY, Chiang WC, Hsieh MJ et al. Quality of audio-assisted versus video-assisted dispatcher-instructed bystander cardiopulmonary resuscitation: A systematic review and meta-analysis. Resuscitation. 2018 Feb;123:77–85. doi: 10.1016/j.resuscitation.2017.12.010

17. Department of Health and Social Care. The future of healthcare: our vision for digital, data and technology in health and care [Internet]. GOV.UK. 2018 [cited 2022 Dec 21]. Available from: https://www.gov.uk/government/publications/the-future-of-healthcare-our-vision-for-digital-data-and-technology-in-health-and-care

18. Gupta T, Gkiousias V, Bhutta MF. A systematic review of outcomes of remote consultation in ENT. Clin Otolaryngol. 2021;46(4):699–719. doi: 10.1111/coa.13768

19. Almathami HKY, Win KT, Vlahu-Gjorgievska E. Barriers and Facilitators That Influence Telemedicine-Based, Real-Time, Online Consultation at Patients' Homes: Systematic Literature Review. J Med Internet Res. 2020 Feb 20;22(2):e16407. doi: 10.2196/16407

20. Walthall H, Schutz S, Snowball J et al. Patients′ and clinicians′ experiences of remote consultation? A narrative synthesis. J Adv Nurs. 2022;78(7):1954–67. doi: 10.1111/jan.15230

21. Armfield NR, Coulthard MG, Slater A et al. The effectiveness of telemedicine for paediatric retrieval consultations: rationale and study design for a pragmatic multicentre randomised controlled trial. BMC Health Serv Res. 2014 Nov 11;14:546. doi: 10.1186/s12913-014-0546-9

22. NIHR. Emergency Medical Services Streaming Enabled Evaluation In Trauma: The SEE - IT trial - NIHR Funding and Awards [Internet]. 2022 [cited 2023 Jan 24]. Available from: https://fundingawards.nihr.ac.uk/award/NIHR130811

23. GoodSAM. GoodSAM [Internet]. GoodSAM Instant Help. 2023 [cited 2023 Jan 24]. Available from: https://www.goodsamapp.org/

24. Weiss DS, Marmar CR. The Impact of Event Scale—Revised. In: Assessing psychological trauma and PTSD. New York, NY, US: The Guilford Press; 1997. p. 399–411.

25. Goldberg D. User's Guide to the General Health Questionnaire. Windsor [Internet]. 1988 [cited 2022 Mar 22]; Available from: https://ci.nii.ac.jp/naid/10016919943/

For peer review only - http://bmjopen.bmj.com/site/about/guidelines.xhtml

26. Endsley MR. Toward a Theory of Situation Awareness in Dynamic Systems. Hum Factors. 1995 Mar 1;37(1):32–64. doi: 10.1518/001872095779049543

27. Croskerry P, Nimmo GR. Better clinical decision making and reducing diagnostic error. J R Coll Physicians Edinb. 2011 Jun;41(2):155–62. doi: 10.4997/JRCPE.2011.208

28. Davis FD. Perceived Usefulness, Perceived Ease of Use, and User Acceptance of Information Technology. MIS Q. 1989;13(3):319–40. doi: 10.2307/249008

29. Bagozzi RP, Davis FD, Warshaw PR. Development and Test of a Theory of Technological Learning and Usage. Hum Relat. 1992 Jul 1;45(7):659–86. doi: 10.1177/001872679204500702

30. Venkatesh V, Davis FD. A Theoretical Extension of the Technology Acceptance Model: Four Longitudinal Field Studies. Manag Sci. 2000;46(2):186–204. doi: 10.1287/mnsc.46.2.186.11926

31. Damschroder LJ, Aron DC, Keith RE et al. Fostering implementation of health services research findings into practice: a consolidated framework for advancing implementation science. Implement Sci. 2009 Aug 7;4(1):50. doi: 10.1186/1748-5908-4-50

32. Qualtrics. Qualtrics XM // The Leading Experience Management Software [Internet]. Qualtrics. 2023 [cited 2023 Feb 1]. Available from: https://www.qualtrics.com/uk/

33. Gale NK, Heath G, Cameron E, Rashid S, Redwood S. Using the framework method for the analysis of qualitative data in multi-disciplinary health research. BMC Med Res Methodol. 2013 Sep 18;13(1):117. doi: 10.1186/1471-2288-13-117

34. ICMJE. ICMJE | Recommendations | Defining the Role of Authors and Contributors [Internet]. Defining the Role of Authors and Contributors. 2023 [cited 2023 Jan 4]. Available from: https://www.icmje.org/recommendations/browse/roles-and-responsibilities/defining-the-role-of-authors-and-contributors.html#two

For peer review only

For peer review only - http://bmjopen.bmj.com/site/about/guidelines.xhtml

SPIRIT

STANDARD PROTOCOL ITEMS: RECOMMENDATIONS FOR INTERVENTIONAL TRIALS

SPIRIT 2013 Checklist: Recommended items to address in a clinical trial protocol and related documents*

Section/item	Item No	Description
**Administrative information**		
Title	1	Descriptive title identifying the study design, population, interventions, and, if applicable, trial acronym
Trial registration	2a	Trial identifier and registry name. If not yet registered, name of intended registry
	2b	All items from the World Health Organization Trial Registration Data Set
Protocol version	3	Date and version identifier
Funding	4	Sources and types of financial, material, and other support
Roles and responsibilities	5a	Names, affiliations, and roles of protocol contributors
	5b	Name and contact information for the trial sponsor
	5c	Role of study sponsor and funders, if any, in study design; collection, management, analysis, and interpretation of data; writing of the report; and the decision to submit the report for publication, including whether they will have ultimate authority over any of these activities
	5d	Composition, roles, and responsibilities of the coordinating centre, steering committee, endpoint adjudication committee, data management team, and other individuals or groups overseeing the trial, if applicable (see Item 21a for data monitoring committee)
**Introduction**		
Background and rationale	6a	Description of research question and justification for undertaking the trial, including summary of relevant studies (published and unpublished) examining benefits and harms for each intervention
	6b	Explanation for choice of comparators
Objectives	7	Specific objectives or hypotheses

For peer review only

Trial design	8	Description of trial design including type of trial (eg, parallel group, crossover, factorial, single group), allocation ratio, and framework (eg, superiority, equivalence, noninferiority, exploratory)

Methods: Participants, interventions, and outcomes

Study setting	9	Description of study settings (eg, community clinic, academic hospital) and list of countries where data will be collected. Reference to where list of study sites can be obtained
Eligibility criteria	10	Inclusion and exclusion criteria for participants. If applicable, eligibility criteria for study centres and individuals who will perform the interventions (eg, surgeons, psychotherapists)
Interventions	11a	Interventions for each group with sufficient detail to allow replication, including how and when they will be administered
	11b	Criteria for discontinuing or modifying allocated interventions for a given trial participant (eg, drug dose change in response to harms, participant request, or improving/worsening disease)
	11c	Strategies to improve adherence to intervention protocols, and any procedures for monitoring adherence (eg, drug tablet return, laboratory tests)
	11d	Relevant concomitant care and interventions that are permitted or prohibited during the trial
Outcomes	12	Primary, secondary, and other outcomes, including the specific measurement variable (eg, systolic blood pressure), analysis metric (eg, change from baseline, final value, time to event), method of aggregation (eg, median, proportion), and time point for each outcome. Explanation of the clinical relevance of chosen efficacy and harm outcomes is strongly recommended
Participant timeline	13	Time schedule of enrolment, interventions (including any run-ins and washouts), assessments, and visits for participants. A schematic diagram is highly recommended (see Figure)
Sample size	14	Estimated number of participants needed to achieve study objectives and how it was determined, including clinical and statistical assumptions supporting any sample size calculations
Recruitment	15	Strategies for achieving adequate participant enrolment to reach target sample size

Methods: Assignment of interventions (for controlled trials)

Allocation:

Sequence generation	16a	Method of generating the allocation sequence (eg, computer-generated random numbers), and list of any factors for stratification. To reduce predictability of a random sequence, details of any planned restriction (eg, blocking) should be provided in a separate document that is unavailable to those who enrol participants or assign interventions
Allocation concealment mechanism	16b	Mechanism of implementing the allocation sequence (eg, central telephone; sequentially numbered, opaque, sealed envelopes), describing any steps to conceal the sequence until interventions are assigned
Implementation	16c	Who will generate the allocation sequence, who will enrol participants, and who will assign participants to interventions
Blinding (masking)	17a	Who will be blinded after assignment to interventions (eg, trial participants, care providers, outcome assessors, data analysts), and how
	17b	If blinded, circumstances under which unblinding is permissible, and procedure for revealing a participant's allocated intervention during the trial
**Methods: Data collection, management, and analysis**		
Data collection methods	18a	Plans for assessment and collection of outcome, baseline, and other trial data, including any related processes to promote data quality (eg, duplicate measurements, training of assessors) and a description of study instruments (eg, questionnaires, laboratory tests) along with their reliability and validity, if known. Reference to where data collection forms can be found, if not in the protocol
	18b	Plans to promote participant retention and complete follow-up, including list of any outcome data to be collected for participants who discontinue or deviate from intervention protocols
Data management	19	Plans for data entry, coding, security, and storage, including any related processes to promote data quality (eg, double data entry; range checks for data values). Reference to where details of data management procedures can be found, if not in the protocol
Statistical methods	20a	Statistical methods for analysing primary and secondary outcomes. Reference to where other details of the statistical analysis plan can be found, if not in the protocol
	20b	Methods for any additional analyses (eg, subgroup and adjusted analyses)
	20c	Definition of analysis population relating to protocol non-adherence (eg, as randomised analysis), and any statistical methods to handle missing data (eg, multiple imputation)

Methods: Monitoring

Data monitoring	21a	Composition of data monitoring committee (DMC); summary of its role and reporting structure; statement of whether it is independent from the sponsor and competing interests; and reference to where further details about its charter can be found, if not in the protocol. Alternatively, an explanation of why a DMC is not needed
	21b	Description of any interim analyses and stopping guidelines, including who will have access to these interim results and make the final decision to terminate the trial
Harms	22	Plans for collecting, assessing, reporting, and managing solicited and spontaneously reported adverse events and other unintended effects of trial interventions or trial conduct
Auditing	23	Frequency and procedures for auditing trial conduct, if any, and whether the process will be independent from investigators and the sponsor

Ethics and dissemination

Research ethics approval	24	Plans for seeking research ethics committee/institutional review board (REC/IRB) approval
Protocol amendments	25	Plans for communicating important protocol modifications (eg, changes to eligibility criteria, outcomes, analyses) to relevant parties (eg, investigators, REC/IRBs, trial participants, trial registries, journals, regulators)
Consent or assent	26a	Who will obtain informed consent or assent from potential trial participants or authorised surrogates, and how (see Item 32)
	26b	Additional consent provisions for collection and use of participant data and biological specimens in ancillary studies, if applicable
Confidentiality	27	How personal information about potential and enrolled participants will be collected, shared, and maintained in order to protect confidentiality before, during, and after the trial
Declaration of interests	28	Financial and other competing interests for principal investigators for the overall trial and each study site
Access to data	29	Statement of who will have access to the final trial dataset, and disclosure of contractual agreements that limit such access for investigators
Ancillary and post-trial care	30	Provisions, if any, for ancillary and post-trial care, and for compensation to those who suffer harm from trial participation

For peer review only - http://bmjopen.bmj.com/site/about/guidelines.xhtml

Dissemination policy	31a	Plans for investigators and sponsor to communicate trial results to participants, healthcare professionals, the public, and other relevant groups (eg, via publication, reporting in results databases, or other data sharing arrangements), including any publication restrictions
	31b	Authorship eligibility guidelines and any intended use of professional writers
	31c	Plans, if any, for granting public access to the full protocol, participant-level dataset, and statistical code
**Appendices**		
Informed consent materials	32	Model consent form and other related documentation given to participants and authorised surrogates
Biological specimens	33	Plans for collection, laboratory evaluation, and storage of biological specimens for genetic or molecular analysis in the current trial and for future use in ancillary studies, if applicable

*It is strongly recommended that this checklist be read in conjunction with the SPIRIT 2013 Explanation & Elaboration for important clarification on the items. Amendments to the protocol should be tracked and dated. The SPIRIT checklist is copyrighted by the SPIRIT Group under the Creative Commons "Attribution-NonCommercial-NoDerivs 3.0 Unported" license.

For peer review only

The SEE-IT Trial: Emergency Medical Services Streaming Enabled Evaluation In Trauma: study protocol for an interventional feasibility randomised controlled trial

Journal:	*BMJ Open*
Manuscript ID	bmjopen-2023-072877.R1
Article Type:	Protocol
Date Submitted by the Author:	10-Mar-2023
Complete List of Authors:	Ollis, Lucie; University of Surrey School of Health Sciences, School of Health Sciences<br>Skene, Simon; University of Surrey, Surrey Clinical Trials Unit<br>Williams, Julia; South East Coast Ambulance Service NHS Foundation Trust; University of Hertfordshire School of Health and Social Work<br>Lyon, Richard; University of Surrey School of Health Sciences; Kent, Surrey & Sussex Air Ambulance<br>Taylor, Cath; University of Surrey School of Health Sciences<br>The SEE-IT Trial Group, The SEE-IT Trial Group; University of Surrey; South East Coast Ambulance Service NHS Foundation Trust
**Primary Subject Heading**:	Emergency medicine
Secondary Subject Heading:	Health services research, Qualitative research
Keywords:	ACCIDENT & EMERGENCY MEDICINE, Telemedicine < BIOTECHNOLOGY & BIOINFORMATICS, Information technology < BIOTECHNOLOGY & BIOINFORMATICS, Feasibility Studies, PRIMARY CARE, TRAUMA MANAGEMENT

The SEE-IT Trial: Emergency Medical Services **S**treaming **E**nabled **E**valuation **I**n **T**rauma: study protocol for an interventional feasibility randomised controlled trial

Lucie Ollis, Simon Skene, Julia Williams, Richard Lyon, and Cath Taylor on behalf of the SEE-IT Trial Group

SEE-IT Trial Group to include:

Kate Bennett-Eastley, Mark Cropley, Heather Gage, Janet Holah, Jill Maben, Carin Magnusson, Craig Mortimer, and Scott Munro.

Corresponding author: Professor Cath Taylor, School of Health Sciences, Kate Granger Building, University of Surrey, Guildford, Surrey, GU2 7XH, cath.taylor@surrey.ac.uk

For peer review only - http://bmjopen.bmj.com/site/about/guidelines.xhtml

ABSTRACT

Introduction

Accurate and timely dispatch of emergency medical resources (EMS) is vital due to limited resources and patients' risk of mortality and morbidity increasing with time. Currently, most UK Emergency Operations Centres (EOCs) rely on audio calls and accurate descriptions of the incident and patients' injuries from lay 999 callers. If dispatchers in the EOCs could see the scene via live video streaming from the caller's smartphone, this may enhance their decision making and enable quicker and more accurate dispatch of EMS. The main aim of this feasibility randomised controlled trial (RCT) is to assess the feasibility of conducting a definitive RCT to assess the clinical and cost effectiveness of using live streaming to improve targeting of EMS.

Methods and analysis

The SEE-IT Trial is a feasibility RCT with a nested process evaluation. The study also has two observational sub-studies: (i) in an EOC who routinely use live streaming to assess the acceptability and feasibility of live streaming in a diverse inner-city population and (ii) in an EOC that does not currently use live streaming to act as a comparator site regarding the psychological wellbeing of EOC staff using vs not using live streaming.

Ethics and dissemination

The study was approved by the Health Research Authority on 23/03/2022 (ref: 21/LO/0912), which included NHS Confidentiality Advisory Group (CAG) approval received on 22/03/2022 (ref: 22/CAG/0003). This manuscript refers to version 0.8 of the protocol (07/11/2022). The trial is registered with the ISRCTN (ISRCTN11449333). The first participant was recruited on 18 June 2022.

The main output of this feasibility trial will be the knowledge gained to help inform the development of a large multi-centre RCT to evaluate the clinical and cost effectiveness of the use of live streaming to aid EMS dispatch for trauma incidents.

For peer review only

Strengths and limitations of this study

- The mixed methods approach to evaluation of feasibility and acceptability uses a combination of routinely collected data, observational data, interviews, and survey methods.
- A comprehensive process evaluation will be undertaken to understand acceptability and impact of using live streaming from the perspective of 999 callers and emergency operations staff.
- The design (testing for a week each month over 6 months) builds consideration of seasonal variation in trauma into the design, whilst enabling time to adapt and improve the protocol as the SEE-IT Trial progresses.
- Recruitment of 999 callers may prove challenging due to the nature of their involvement in incidents; and investigating any impact of wider diversity factors is dependent on the characteristics of callers during the trial periods and we may not have sufficient data for this.The Ambulance Trust where live streaming will be tested uses NHS Pathways (as used in half of Ambulance Trusts in UK); the SEE-IT Trial will give us insight into how live streaming would work with this dispatch system but may not extrapolate fully to areas that use other systems for triage/decision support.

For peer review only

INTRODUCTION

Background and Rationale

Emergency medical services (EMS) provide emergency medical care at the scene of incidents before patients are conveyed to hospital (e.g., blood transfusions and emergency anaesthesia). To save lives and prevent long term disability, timely and accurate dispatch of pre-hospital resources is critical (1). Evidence suggests trauma patients should receive definitive care within one hour of the incident (The 'Golden Hour'), otherwise the risk of morbidity and mortality increases significantly (2). Where severe injury is suspected by EMS (e.g., major trauma), specialist paramedics in critical care and/or Helicopter Emergency Medical Services (HEMS) may be dispatched to the scene to care for the patient and/or convey the patient to hospital.

When a 999 call is made in the UK, the dispatchers in the Emergency Operations Centre (EOC) need to decide which resources to send to the scene by gathering information about what has happened and the clinical state of the patient(s). The dispatchers rely on 999 callers and other bystanders to accurately relay essential information (3). However, lay public 999 callers can sometimes provide inaccurate information due to language barriers, subjectivity, the emotional impact of being at the scene and limited medical training or knowledge (3,4). Dispatch response is often recognised as a weak link in the EMS chain (5), often involving either under-resourcing or over-resourcing (6–9). Previous studies have shown that up to 50% of HEMS (air ambulance) dispatch and 25% of road ambulance dispatch for suspected trauma patients are not appropriate (6–9).

It is vital that EMS are being dispatched to incidents where they are needed due to finite and often limited resources. If dispatchers were able to see the patient and scene via live video streaming through a 999 callers' phone camera, it could be easier to make more accurate and timely decisions about dispatch of EMS (4). There is evidence that video-assisted CPR has the potential to improve patient outcomes (10–16), but there is a lack of research evidence to support the use of live video streaming for EMS dispatch for trauma.

NHS policies and the Department of Health and Social Care actively encourage the use of innovative technologies to improve patient and health care outcomes (17). The benefits of using video in other healthcare settings is growing, e.g. for remote healthcare consultations (18–21), but evidence is sparse in relation to use in pre-hospital settings, despite being used routinely in some ambulance services. There is some limited evidence of the benefits and

For peer review only - http://bmjopen.bmj.com/site/about/guidelines.xhtml

acceptability of live streaming to aid HEMS dispatch (4), but its impact on clinical or economic outcomes has not yet been evaluated. Also, the psychological impact of dispatchers viewing the scene and impact on 999 callers has not been investigated for exposure to trauma.

Objectives and research questions

Main research question: Is it feasible to conduct a future randomised controlled trial (RCT) to assess the clinical and cost effectiveness of using GoodSAM live video streaming to improve targeting of emergency medical resources? The overall aim of this research is to assess the feasibility of implementing and evaluating GoodSAM live streaming in a definitive RCT.

The objectives are:

(i) To obtain data required to inform the design of a subsequent RCT (e.g., event rate, screening rate, effect size/precision for outcomes, health economic data).
(ii) To test trial processes including randomisation and data collection methods.
(iii) To conduct a nested process evaluation to test the acceptability and feasibility of using GoodSAM live streaming from provider and public perspectives (e.g., training, video feasibility, video acceptability, psychological harm to 999 callers and/or dispatch staff).

Further research questions relating to these objectives can be found in the study protocol published on the National Institute for Health and Care Research (NIHR) study webpage (22).

METHODS AND ANALYSIS

Trial design and study setting

The SEE-IT Trial is a feasibility RCT with a nested process evaluation in an EOC in South East England (South East Coast Ambulance Service NHS Trust). This study includes two observational sub-studies (i) in an EOC who routinely use live streaming to assess acceptability and feasibility of use of live streaming in a diverse inner-city population (London Ambulance Service NHS Trust) and (ii) in an EOC that does not currently use live streaming to act as a comparator site regarding the psychological wellbeing of EOC staff using vs not using live streaming (East of England Ambulance Service NHS Trust). In addition, eleven NHS hospital trusts covered by the main trial site will be involved in identifying and consenting patients for access to their medical records including: University

For peer review only

Hospital Southampton NHS Foundation Trust; St George's University Hospitals NHS Foundation Trust; Medway NHS Foundation Trust; Surrey and Sussex Healthcare NHS Trust; Maidstone and Tunbridge Wells NHS Trust; East Sussex Healthcare NHS Trust; East Kent Hospitals University NHS Foundation Trust; Dartford and Gravesham NHS Trust; King's College University Hospital NHS Trust; Royal Surrey County Hospital NHS Foundation Trust and University Hospitals Sussex NHS Foundation Trust.

The study was planned to run for 18 months from 1st October 2021 to 31st March 2023. Pressures on the NHS and ambulance services meant the start of the trial period was delayed 4 months to June 2022. Therefore, the overall study end date is now 31st July 2023. There were nine amendments made to the protocol during the feasibility study, outlined in the protocol on the NIHR study webpage (22).

Eligibility criteria

This study includes three types of participants; trauma patients, lay public 999 callers and EOC staff. See tables 1 and 2 for inclusion and exclusion criteria respectively.

Table 1: Participant inclusion criteria

Participant type	Main feasibility trial	Inner-city observational sub-study	Staff wellbeing sub-study
**Trauma patients**	All trauma patients during the six trial observation weeks who are the subject of 999 calls involving major trauma, judged by the HEMS dispatcher and/or Critical Care Paramedics (CCPs) as likely to require enhanced dispatch.	All trauma patients during observed shifts that involve trauma and are screened by HEMS dispatchers or critical care advanced paramedic practitioner dispatchers (APPs) who attempt to use GoodSAM during the call.	N/A
**Lay public 999 callers**	999 callers during the six trial weeks where the incident involves major trauma (defined as per above).	All 999 callers during observed shifts that involve trauma and are screened by HEMS dispatchers or critical care APPs who attempt to use GoodSAM during the call.	N/A
**EOC staff**	All CCPs, HEMS dispatchers, and research paramedics.	All HEMS dispatchers and critical care APPs.	All CCPs and HEMS dispatchers.

Note: CCPs in the main feasibility trial site and APPs in the inner-city observational sub study are the ways different Ambulance Trusts describe specialist paramedics in critical care.

For peer review only - http://bmjopen.bmj.com/site/about/guidelines.xhtml

Table 2: Participant exclusion criteria

Participant type	Main feasibility trial	Inner-city observational sub-study	Staff wellbeing sub-study
**Trauma patients**	Any emergencies of a suspected medical origin (e.g., heart attack or stroke).	Any emergencies of a suspected medical origin (e.g., heart attack or stroke).	N/A
**Lay public 999 callers**	Calls will be excluded where: (i) 999 caller is not at the scene; (ii) 999 call originates from a landline; (iii) 999 call originates from another emergency service e.g. police or fire; (iv) 999 calls where resource (excluding community first responder, CFR) will arrive on scene before live streaming could be activated; (v) 999 call ended before transfer for activation of live streaming; (vi) 999 calls where another incident takes priority; and (vii) calls where clinical acuity is found to be lower than the threshold for the study (not major trauma).<br>All callers identified by the dispatchers as a child caller (under 16 years old) and those who select they are under 16 on the 999-caller survey will be excluded.	All callers identified by the dispatchers as a child caller (under 16 years old) and those who select they are under 16 on the 999-caller survey will be excluded.	N/A
**EOC staff**	EOC staff not mentioned in the inclusion criteria	EOC staff not mentioned in the inclusion criteria	EOC staff not mentioned in the inclusion criteria

Intervention and randomisation

999 calls in the main trial site during the six observation weeks (up to 42 days; 84 shifts) will be randomised 1:1 to working shift intervention or standard care. Control shifts will follow standard care ambulance dispatch protocol with a 999 caller using a telephone (voice only) and the call taker using NHS Pathways ambulance dispatch tool.

For peer review only - http://bmjopen.bmj.com/site/about/guidelines.xhtml

In the intervention arm, a live streaming technology called GoodSAM Instant-on-Scene™ (23) will be tested. The technology allows a 999 caller to stream live video footage from the scene of an incident to the EOC. 999 calls allocated to intervention will initially follow the standard NHS Pathways dispatch protocol until ambulance dispatch prioritisation has been determined. EMS will be dispatched as normal, without delay. The call handler in the EOC will then transfer the caller to the HEMS dispatcher or Critical Care Paramedic (CCP) and live streaming will be activated. EMS resource allocation may be adjusted following this.

If at any point the dispatcher feels they do not want to view the scene (e.g., due to psychological impact of viewing the scene) or it is not safe to continue, the 999 caller will be thanked for their help and the call will be ended. The dispatchers will be provided with training and guides outlining the trial processes to ensure adherence to intervention and study protocols.

Outcomes

Primary outcomes

The primary outcome is the decision regarding the feasibility of undertaking a definitive RCT based primarily on meeting the specified progression criteria (see table 3).

Table 3: Progression criteria from feasibility RCT to future RCT

GREEN; proceed to definitive study - GO	AMBER; consider protocol amendments to improve criteria	RED; do not proceed to main trial - STOP
≥70% of callers with smartphones agreeing and able to activate live streaming	≥50% of callers with smartphones agreeing and able to activate live streaming	<50% of callers with smartphones agreeing and able to activate live streaming
≥50% of requests to activate live streaming resulting in footage being viewed	≥30% but <50% of requests to activate live streaming resulting in footage being viewed	<30% of requests to activate live streaming resulting in footage being viewed
Air Ambulance (HEMS) stand-down rate reducing by ≥10% and/or change in dispatch decision as a result of live streamed footage confirmed as being appropriate in ≥10% cases	Air Ambulance stand-down rate reducing by ≥5% and/or change in dispatch decision as a result of live streamed footage confirmed as being appropriate in ≥5% cases	No change in Air Ambulance stand-down rate and/or change in dispatch decision as a result of live streamed footage

For peer review only - http://bmjopen.bmj.com/site/about/guidelines.xhtml

Rates of psychological harm (based on the survey measures) not significantly greater in 999 callers using live streaming compared to those not; and no significant difference in change to psychological harm over time in staff (CCPs, HEMS dispatchers, Research Paramedics) compared to change in staff in a comparison EOC not using live streaming/streamed from scene footage	-	Evidence of significantly greater harm in either 999 callers or EOC staff using live streaming compared to EOC not using live streaming.

In addition to the quantitative progression criteria described in table 3, the research team will consider qualitative data collected as part of this study (e.g., interviews, observations, and free text questions in surveys) when reviewing progression to a definitive RCT.

Secondary outcomes

Speed of appropriate EMS dispatch: speed will be measured using time-stamped data from start of 999 calls to appropriate deployment with judgement of 'appropriateness' to be based on algorithms developed through expert consensus (see below) and using medical records data up to 3 months post-incident where consent is provided; Stand-down rate (de-escalation); missed jobs (e.g. not prioritised for HEMS/CCP despatch, either due to lack of resource or inappropriate prioritisation); requests for further ambulance resources from scene. Psychological harm will be assessed using two validated scales (24, 25) pre and post-trial in 999 callers from both arms and staff viewing the footage (including staff in comparison EOC).

The appropriateness of dispatch will be determined based on algorithms developed through expert consensus before the trial starts. Appropriateness is defined as the need for intervention and/or transport by the response vehicle/staff dispatched to scene (e.g. for HEMS dispatch that a critical care intervention and/or transportation by helicopter was required (9)). A binary outcome decision tree will be developed with the reasons for appropriate/inappropriate dispatch categorised and agreed (inappropriate dispatch will be further categorised as being 'over' or 'under' resourced).

Participant timeline

There will be six trial weeks, one week a month from June to November 2022. Each trial week will include up to 14 randomised shifts (day and night). EOC staff in the main trial site and staff wellbeing sub study will be invited to participate in the staff survey pre- and post-

For peer review only - http://bmjopen.bmj.com/site/about/guidelines.xhtml

trial period. Staff in the main trial site will be invited to partake in interviews through the survey. 999 callers in the main trial site will be sent a text message after the eligible incident inviting them to participate in a survey 6-8 weeks post incident and a subsequent interview. Patients will be approached for consent for their medical records to be accessed up to 3 months after the incident has occurred.

In the observational sub-study, EOC staff will be observed using GoodSAM live streaming over a 3-month period and invited to partake in interviews. 999 callers will be invited to partake in the survey within 1 week of the incident they called 999 for and subsequently invited to partake in an interview about their experiences of using GoodSAM live streaming.

Sample size

We estimate there will be approximately 250 trauma incidents (including 300 patients) over the six observation weeks (125 allocated to intervention), which will allow for an estimate of true event rate within precision of +/-0.75 events per day; and allow estimation of speed to appropriate response with a standard error of <5%. These sample size estimations are based on convenience sampling over the six defined trial observation weeks. We estimate there will be approximately 350 lay public 999 callers (250 from the main trial site and 100 from the observational sub-study) and up to 202 EOC staff (86 from the main trial site, 30 from the observational sub-study and 86 from the third EOC).

Recruitment: Identification and consent

Trauma patients

Trauma patients will be identified by Research Paramedics (RPs), using the eligibility criteria. RPs will observe all trial shifts. For eligible calls in the main trial site, the Computer Aided Dispatch (CAD) number(s) relating to the incident will be recorded by the RP as the identifier to share with the conveying hospital to approach for consent. Informed consent will be sought from either the patient, their consultee or the patient's parent or guardian (either in the hospital before discharge or via telephone, email, or post) in all hospital trusts included in the trial (trauma units and major trauma centres covered by the EOC). RPs may consent patients in person with assistance from the hospital to identify the patient(s). Approval has been granted by the Confidentiality Advisory Group (Section 251 application) to access medical records for casualties who die before consent can be sought (and where there is not a

For peer review only - http://bmjopen.bmj.com/site/about/guidelines.xhtml

power of attorney in place for that individual). National data opt-out status will be checked for all deceased patients.

999 callers

In the main feasibility site, the RP will determine eligibility of the 999 callers and prompt the dispatchers to send a text message via the CAD, inviting the 999 caller to participate in a survey about their experience of calling 999 for the incident. The text message will include a link to the PIS, consent form and contact details for the research team. A reminder text message will be sent to 999 callers within one week of the incident. Those who participate in the survey will be invited to partake in an interview and selected to represent a range in demographics (age, ethnicity, gender), type of incident they witnessed and evidence or not of psychological harm from survey responses.

In the inner-city observational sub-study, at the end of the call (once resources have been dispatched) 999 callers will be asked for permission to store their name and number to share with the researcher to be invited to participate in the study. Those who consent will initially be contacted by phone so the researcher can explain the study and ask their permission to send them further details about the study via text message or email. The researcher will then send a text message or email with a link to the PIS and consent form, which once completed will re-direct to the survey. Reminder texts/emails will be sent up to one week after the incident. Only those who participate in the survey will be invited to partake in an interview.

EOC staff

In the main trial site, the use of live streaming has been approved at organisation level and thereby staff are not individually consented to use live streaming but instead will be given the opportunity to opt out of trial shifts. They will otherwise be required to follow organisational protocols if they are working on a trial shift. In the observational sub-study, HEMS dispatchers and critical care APPs will consent on an individual basis to being observed using live streaming. The researcher will contact staff to invite them to participate in an interview about the use of live streaming in their organisation. The local PI at both EOCs participating in the staff survey will share the staff survey link with all eligible staff pre-trial. All staff who enter their email address to complete the post-trial survey will be emailed a link to the survey post-trial. Those who participate in the staff survey at the main trial site will be invited to partake in an interview about their views and experiences of live streaming; selected to include different types of staff e.g., age, gender, and experience.

For peer review only - http://bmjopen.bmj.com/site/about/guidelines.xhtml

Assignment of intervention

Working shifts are randomised 1:1 to intervention (GoodSAM) or standard ambulance care using a minimisation algorithm to ensure balance between weekday/weekend (Mon-Thurs vs Fri-Sun) and daytime/night-time shifts (0600-1800 vs 1800-0600).

A small number of shifts in the final observation week will be used to test the feasibility of individual call randomisation using a pre-prepared randomisation list. All randomisations will be undertaken by a statistician at Surrey CTU to ensure the allocation is independent of the research team.

Data collection methods

Observations of EOC processes

An experienced researcher will observe EOC processes, decision making processes, fidelity to the protocol and impact of contextual variability (e.g., time of day, weather) during the trial weeks in the main trial site (3-6 hours for 3 days in each observation week). In the observational sub-study, a second experienced researcher will observe up to 24 shifts over a 3-month period. Observational data will be recorded using fieldnotes capturing both descriptive and conceptual observations/interpretations.

The observational and interview data collection and analysis will be underpinned by theories informing the proposed mechanisms of effect e.g. Situational Awareness, SA (26); Decision Making, DM (27); the implementation of a new technology, TAM (28–30); and Consolidated Framework for Implementation Research, CFIR (31). Events will be observed in real time and the use of live streaming will be understood in the context of how the use of visual technology impacts on staff assessment of situations and action taken (SA and DM), as well as the factors influencing how people relate to the introduction of a new technology (TAM). Equally, in situations where live streaming is not used, theories of SA and DC will inform observations in context.

Decision making data and clinical data from trauma patients

During the trial weeks, the RPs will collect data live during incidents including the CAD number; information about decision making; the patients' name, age, and sex (if available); details of the trauma incident (e.g., single/multiple casualties; general nature of incident e.g., road traffic collision/assault); dispatch decisions; and the use of live streaming e.g., quality of

For peer review only - http://bmjopen.bmj.com/site/about/guidelines.xhtml

sound/picture. This information will be recorded on a piloted, study-specific proforma completed at the time of the incident. Further details about EMS dispatch (including speed to appropriate dispatch) and time stamped data for each incident will be accessed by RPs after the incident.

Following consent to access medical records (process described in recruitment section), the RPs will extract relevant information from patients' medical records about the injuries sustained and the treatments received (up to 3 months post-incident). Using the expert panel algorithms, the RPs will rate the appropriateness of dispatch. A random 10% sample of incidents from intervention and control calls will be independently reviewed by the expert panel using the same data, to assess reliability.

999 callers

999 callers in the main trial site will be invited to complete surveys via Qualtrics online survey software (32) 6-8 weeks post incident to assess psychological harm and semi-structured interviews regarding experiences and acceptability of the use of live streaming. The questionnaire will include demographic questions, The Impact of Events Scale - Revised (24), The General Health Questionnaire-12 item version (25) and questions about acceptability and experience of live streaming.

In the inner-city observational sub-study, 999 callers will be invited to complete surveys via Qualtrics (32) up to one week after the incident. The questionnaire will include a demographic questionnaire and questions about their experiences of using live streaming during their recent 999 call e.g., ease of use, usefulness. Those who take part in the questionnaire will be invited to participate in an interview regarding experiences and acceptability of using live streaming.

999 callers at both sites who participate in the survey will be offered the choice of a £10 shopping voucher or £10 donation to the local air ambulance charity. A second voucher or donation will be offered to those who complete an interview.

EOC staff survey and interviews

Before the trial starts, EOC staff at the main trial site and staff wellbeing sub-study will be emailed by the local PI including the PIS, consent form and a link to the online survey via Qualtrics (32). The survey will include a demographic questionnaire, The Impact of Events Scale - Revised (24), The General Health Questionnaire-12 item version (25) and questions

For peer review only - http://bmjopen.bmj.com/site/about/guidelines.xhtml

about live streaming. Those who complete both pre- and post-trial questionnaires will be entered into a prize draw for one of five £50 retail vouchers (five available at each site). In the main trial site, approximately 12-18 staff will be invited to participate in an interview and will be paid overtime rates for their participation. Staff in the observational sub-study who take part in an interview will be compensated for their time with a £45 Amazon voucher. The different methods for reimbursing time have been requested by the respective organisations.

Data management

A detailed data management plan has been produced outlining the processes for completion, transfer, and storage of study data in line with the Ambulance Trust and University policies, the requirements of the Sponsor and GDPR. The data management plan will be shared with all those responsible for data collection on the trial and will form the basis of a Data Protection Impact Assessment (DPIA) and data processing agreements between the parties.

Statistical methods and data analysis

Quantitative analysis

A full Statistical Analysis Plan (SAP) will be reviewed by key members of the research team and approved by the Steering Committee before analysis is undertaken.

As this is a feasibility study, statistical analysis will focus on providing estimates and confidence intervals of key rates such as the number of eligible calls per day, uptake of video intervention and timing and appropriateness of dispatch to inform a subsequent RCT. Outcome measure will be summarised by arm and estimates reported with confidence intervals to inform future sample size calculations.

The SAP will outline any expected exploratory analysis of outcomes that may be useful to inform a subsequent RCT including any signal of potential efficacy, but it is noted that the study is not powered for this and so any interpretation would be limited to the direction and magnitude of any effect. Missing data (including outcome data) will be summarised, but all observed data will be included in any analysis according to the randomised allocation following the intention-to-treat principle.

Qualitative analysis

The process evaluation will include analysis of live streaming usage data; observations of EOC processes; and interviews with staff and 999 callers. All interviews in the main trial site

For peer review only - http://bmjopen.bmj.com/site/about/guidelines.xhtml

and observational sub-study will be digitally recorded and transcribed verbatim. Once the transcripts have been checked for accuracy, recordings will be deleted. Transcripts will be analysed thematically using the Framework Method (33). Data will be analysed concurrent to its collection where possible and will inform the topic guides for interviews.

Health economic analysis

A health economic analysis will be undertaken from NHS and societal perspectives to inform the design of a full economic evaluation within the future RCT. The primary purpose of the health economic analysis is to assess the feasibility of gathering data on the resource implications, costs, and effects of the dispatch decisions under standard care and when adjusted for the live streaming intervention.

Data Monitoring

A data monitoring committee was not required as this study is a feasibility study and there is no interim analysis.

Harms

The study teams and participating staff at EOCs will inform the Chief Investigator and/or Sponsor as soon as they are aware of a possible serious breach that could impact the safety, or physical or mental integrity of subjects/participants in the study, so that the Sponsor can fulfil its regulatory and oversight requirements. Other deviations from the protocol will be recorded and dealt with appropriately.

Auditing

The research team at each study site will be responsible for ensuring the accuracy and quality of data collection and data entry. Several data audit checks will be conducted by the RPs prior to transfer of the study database to the university and analysis to ensure accurate data recording.

Committees

Steering Committee (SC)

The SC will monitor progress against milestones, spend against budget, provide advice where necessary, promote the project, facilitate communication between organisations with

For peer review only - http://bmjopen.bmj.com/site/about/guidelines.xhtml

stakeholders, and help maximise dissemination and impact of findings. Membership will be independently appointed and NIHR-approved.

Project Advisory Group (PAG)

The project will be supported by a PAG, chaired by the PPIE lead who will also chair a separate but inter-connected PPIE Group. The PAG will meet up to 4 times over the course of the study to coincide with key timepoints in the project and provide a forum for input and support regarding the data collection, analysis, and production of outputs and dissemination.

Patient and Public Involvement and Engagement Group (PPIE)

The PPIE group will be formed of up to five lay representatives and be chaired by a lay public PPIE lead. We will ask for their assistance with developing patient-facing materials and input into strategic and management decisions. We will involve the PPIE group during data analysis, through sense-checking emerging results and considering how to interpret findings. Finally, we will work with the PPIE group to determine the most effective ways of dissemination, using a variety of approaches from journal publications and conference presentations, through to use of social media sites and a short film.

ETHICS AND DISSEMINATION

Research Ethics Approval

Favourable ethical opinion was granted by London – Camden & King's Cross Research Ethics Committee (reference 21/LO/0912).

Protocol amendments

Amendments to the protocol will be updated on the ISRCTN registry, uploaded to the NIHR project webpage, and shared with all study sites and key members of the research team.

Confidentiality

All trial data will be anonymised prior to transfer to the University for analysis. Qualitative data (interviews, observational field notes) will be anonymised as soon as possible after collection and only anonymised data will be analysed. Any quotes used to illustrate findings will be unidentifiable to the source.

Declaration of Interests

None declared.

For peer review only - http://bmjopen.bmj.com/site/about/guidelines.xhtml

For peer review only

Access to Data

Direct access will be granted to authorised representatives from the Sponsor, host institution and regulatory authorities to permit trial-related monitoring, audits, and inspections, in line with participant consent. Access to the final dataset will be restricted to members of the research team. The data will be kept securely and in a pseudo-anonymised format to protect personal sensitive data from being associated with any individual or participant.

Dissemination

The main output of this feasibility RCT will be the knowledge gained regarding the acceptability and feasibility of using live streaming in practice and the learning about data collection and research processes to inform the development of a larger multi-centre RCT. Findings will be shared with key stakeholders (e.g., policy makers, commissioners, providers, emergency response staff, patients and public, academic audiences) by a wide variety of means, including journal publications, conference presentations, summary reports, media releases, the project webpage (www.surrey.ac.uk/seeitstudy), social media and a short film. Authorship of outputs will be decided using the International Committee of Medical Journal Editors criteria (ICMJE (34)) and has been agreed with all members of the research team. The full dissemination policy can be found in the published protocol on the NIHR webpage (22).

ADMINISTRATIVE INFORMATION

Author contributions: The original protocol was drafted by CT and SS, with significant contributions from JW and RL, and based on the original grant proposal contributed to by all co-applicants. The manuscript was drafted by LO, and critically reviewed by all other authors named in the SEE-IT Trial Group. All authors approved the final version of the protocol.

Trial sponsor: The trial Sponsor is the University of Surrey Research Integrity and Governance Office (reference SPON 2021 22 FHMS, rigo@surrey.ac.uk, 01483 683490) with trial management activities conducted by the University of Surrey Clinical Trials Unit.

Funding statement: This study/project is funded by the NIHR Health and Social Care Delivery Research (NIHR130811, Injuries and Accidents, https://fundingawards.nihr.ac.uk/award/NIHR130811). The views expressed are those of the author(s) and not necessarily those of the NIHR or the Department of Health and Social Care. The project is also supported by the Clinical Research Network and has received favourable opinion from the HRA (21/LO/0912). The trial sponsor and funding body contributed to the

For peer review only - http://bmjopen.bmj.com/site/about/guidelines.xhtml

design of this study but will not have any role during its execution, analysis, interpretation if the data, writing of the report or the decision to submit the report for publication.

Competing interests: None declared.

Acknowledgments: We would like to thank all have supported this study within Air Ambulance Charity Kent, Surrey, Sussex; South East Coast Ambulance Service NHS Foundation Trust; London Ambulance Service; East of England Ambulance Service; and the major trauma centres and units in the region. In addition, we would like to thank our PPIE Group; Project Advisory Group; Steering Committee and Expert Panel members for their valuable contributions. Finally, we would like to thank Oliver Fitzgerald, Fon Yan Li, Andrew Fooks, Caroline Cripps, and Anthony Coe for contributing to data collection; Rachael Cooke for the project administrative support; and Matthew Glover for contributing to the health economic methods.

For peer review only

For peer review only - http://bmjopen.bmj.com/site/about/guidelines.xhtml

REFERENCES

1. Duke M, Tatum D, Sexton K et al. When Minutes Fly by: What is the True "Golden Hour" for Air Care? Am Surg. 2018 Jun;84(6):862–7. doi: 10.1177/000313481808400633

2. Pham H, Puckett Y, Dissanaike S. Faster on-scene times associated with decreased mortality in Helicopter Emergency Medical Services (HEMS) transported trauma patients. Trauma Surg Acute Care Open. 2017 Oct 1;2(1):e000122. doi: 10.1136/tsaco-2017-000122

3. Carroll LN, Calhoun RE, Subido CC et al. Serving Limited English Proficient Callers: A Survey of 9-1-1 Police Telecommunicators. Prehospital Disaster Med. 2013 Jun;28(3):286–91. doi: 10.1017/S1049023X13000265

4. ter Avest E, Lambert E, de Coverly R et al. Live video footage from scene to aid helicopter emergency medical service dispatch: a feasibility study. Scand J Trauma Resusc Emerg Med. 2019 Dec;27(1):55. doi: 10.1186/s13049-019-0632-4

5. Schellhaaß A, Popp E. Air rescue: current significance and practical issues. Anaesthesist. 2014 Dec 1;63(12):971–80; quiz 981–2. doi: 10.1007/s00101-014-2356-1

6. Eaton G, Raitt J, Brown S et al. How appropriate are the data produced by NHS Pathways for identifying suitable cases for involvement of enhanced care teams? Br Paramed J. 2017 Dec 1;2(3):16–23. doi: 10.29045/14784726.2017.2.3.16

7. Palazzo FF, Warner OJ, Harron M et al. Misuse of the London ambulance service: How much and why? Emerg Med J. 1998 Nov 1;15(6):368–70. doi: 10.1136/emj.15.6.368

8. Wilmer I, Chalk G, Davies GE et al. Air ambulance tasking: mechanism of injury, telephone interrogation or ambulance crew assessment?: Table 1. Emerg Med J. 2015 Oct;32(10):813–6. doi: 10.1136/emermed-2013-203204

9. Munro S, Joy M, de Coverly R, et al. A novel method of non-clinical dispatch is associated with a higher rate of critical Helicopter Emergency Medical Service intervention. Scand J Trauma Resusc Emerg Med. 2018 Sep 25;26(1):84. doi: 10.1186/s13049-018-0551-9

10. Kim GW, Lee DK, Kang BR et al. A multidisciplinary approach for improving the outcome of out-of-hospital cardiac arrest in South Korea. Eur J Emerg Med Off J Eur Soc Emerg Med. 2020 Feb;27(1):46–53. doi: 10.1097/MEJ.0000000000000612

11. Johnsen E, Bolle SR. TO SEE OR NOT TO SEE—Better dispatcher-assisted CPR with video-calls? A qualitative study based on simulated trials. Resuscitation. 2008 Sep;78(3):320–6. doi: 10.1016/j.resuscitation.2008.04.024

12. Huang HK, Chen HH, Chen YL et al. A Novel Assessment Using a Panoramic Video Camera of Resuscitation Quality in Patients following Out-of-Hospital Cardiac Arrest. Prehospital Emerg Care Off J Natl Assoc EMS Physicians Natl Assoc State EMS Dir. 2022 Jan 25;1–4. doi: 10.1080/10903127.2021.2015025

13. Lee HS, You K, Jeon JP et al. The effect of video-instructed versus audio-instructed dispatcher-assisted cardiopulmonary resuscitation on patient outcomes following out of

For peer review only

hospital cardiac arrest in Seoul. Sci Rep. 2021 Jul 30;11(1):15555. doi: 10.1038/s41598-021-95077-5

14. Linderoth G, Rosenkrantz O, Lippert F et al. Live video from bystanders' smartphones to improve cardiopulmonary resuscitation. Resuscitation. 2021 Nov;168:35–43. doi: 10.1016/j.resuscitation.2021.08.048

15. Linderoth G, Møller TP, Folke F, Lippert FK, Østergaard D. Medical dispatchers' perception of visual information in real out-of-hospital cardiac arrest: a qualitative interview study. Scand J Trauma Resusc Emerg Med. 2019 Jan 25;27(1):8. doi: 10.1186/s13049-018-0584-0

16. Lin YY, Chiang WC, Hsieh MJ et al. Quality of audio-assisted versus video-assisted dispatcher-instructed bystander cardiopulmonary resuscitation: A systematic review and meta-analysis. Resuscitation. 2018 Feb;123:77–85. doi: 10.1016/j.resuscitation.2017.12.010

17. Department of Health and Social Care. The future of healthcare: our vision for digital, data and technology in health and care [Internet]. GOV.UK. 2018 [cited 2022 Dec 21]. Available from: https://www.gov.uk/government/publications/the-future-of-healthcare-our-vision-for-digital-data-and-technology-in-health-and-care

18. Gupta T, Gkiousias V, Bhutta MF. A systematic review of outcomes of remote consultation in ENT. Clin Otolaryngol. 2021;46(4):699–719. doi: 10.1111/coa.13768

19. Almathami HKY, Win KT, Vlahu-Gjorgievska E. Barriers and Facilitators That Influence Telemedicine-Based, Real-Time, Online Consultation at Patients' Homes: Systematic Literature Review. J Med Internet Res. 2020 Feb 20;22(2):e16407. doi: 10.2196/16407

20. Walthall H, Schutz S, Snowball J et al. Patients′ and clinicians′ experiences of remote consultation? A narrative synthesis. J Adv Nurs. 2022;78(7):1954–67. doi: 10.1111/jan.15230

21. Armfield NR, Coulthard MG, Slater A et al. The effectiveness of telemedicine for paediatric retrieval consultations: rationale and study design for a pragmatic multicentre randomised controlled trial. BMC Health Serv Res. 2014 Nov 11;14:546. doi: 10.1186/s12913-014-0546-9

22. NIHR. Emergency Medical Services Streaming Enabled Evaluation In Trauma: The SEE - IT trial - NIHR Funding and Awards [Internet]. 2022 [cited 2023 Jan 24]. Available from: https://fundingawards.nihr.ac.uk/award/NIHR130811

23. GoodSAM. GoodSAM [Internet]. GoodSAM Instant Help. 2023 [cited 2023 Jan 24]. Available from: https://www.goodsamapp.org/

24. Weiss DS, Marmar CR. The Impact of Event Scale—Revised. In: Assessing psychological trauma and PTSD. New York, NY, US: The Guilford Press; 1997. p. 399–411.

25. Goldberg D. User's Guide to the General Health Questionnaire. Windsor [Internet]. 1988 [cited 2022 Mar 22]; Available from: https://ci.nii.ac.jp/naid/10016919943/

For peer review only - http://bmjopen.bmj.com/site/about/guidelines.xhtml

26. Endsley MR. Toward a Theory of Situation Awareness in Dynamic Systems. Hum Factors. 1995 Mar 1;37(1):32–64. doi: 10.1518/001872095779049543

27. Croskerry P, Nimmo GR. Better clinical decision making and reducing diagnostic error. J R Coll Physicians Edinb. 2011 Jun;41(2):155–62. doi: 10.4997/JRCPE.2011.208

28. Davis FD. Perceived Usefulness, Perceived Ease of Use, and User Acceptance of Information Technology. MIS Q. 1989;13(3):319–40. doi: 10.2307/249008

29. Bagozzi RP, Davis FD, Warshaw PR. Development and Test of a Theory of Technological Learning and Usage. Hum Relat. 1992 Jul 1;45(7):659–86. doi: 10.1177/001872679204500702

30. Venkatesh V, Davis FD. A Theoretical Extension of the Technology Acceptance Model: Four Longitudinal Field Studies. Manag Sci. 2000;46(2):186–204. doi: 10.1287/mnsc.46.2.186.11926

31. Damschroder LJ, Aron DC, Keith RE et al. Fostering implementation of health services research findings into practice: a consolidated framework for advancing implementation science. Implement Sci. 2009 Aug 7;4(1):50. doi: 10.1186/1748-5908-4-50

32. Qualtrics. Qualtrics XM // The Leading Experience Management Software [Internet]. Qualtrics. 2023 [cited 2023 Feb 1]. Available from: https://www.qualtrics.com/uk/

33. Gale NK, Heath G, Cameron E, Rashid S, Redwood S. Using the framework method for the analysis of qualitative data in multi-disciplinary health research. BMC Med Res Methodol. 2013 Sep 18;13(1):117. doi: 10.1186/1471-2288-13-117

34. ICMJE. ICMJE | Recommendations | Defining the Role of Authors and Contributors [Internet]. Defining the Role of Authors and Contributors. 2023 [cited 2023 Jan 4]. Available from: https://www.icmje.org/recommendations/browse/roles-and-responsibilities/defining-the-role-of-authors-and-contributors.html#two

For peer review only

For peer review only - http://bmjopen.bmj.com/site/about/guidelines.xhtml

STANDARD PROTOCOL ITEMS: RECOMMENDATIONS FOR INTERVENTIONAL TRIALS

SPIRIT 2013 Checklist: Recommended items to address in a clinical trial protocol and related documents*

Section/item	Item No	Description	Location in manuscript (page number(s), lines)
**Administrative information**			
Title	1	Descriptive title identifying the study design, population, interventions, and, if applicable, trial acronym	Page 1, lines 1-3
Trial registration	2a	Trial identifier and registry name. If not yet registered, name of intended registry	Page 2, lines 22-23
	2b	All items from the World Health Organization Trial Registration Data Set	N/A in trial registry
Protocol version	3	Date and version identifier	Page 2, lines 21-22
Funding	4	Sources and types of financial, material, and other support	Page 17, lines 26-31
Roles and responsibilities	5a	Names, affiliations, and roles of protocol contributors	Page 17, lines 20-23
	5b	Name and contact information for the trial sponsor	Page 17, lines 24-26
	5c	Role of study sponsor and funders, if any, in study design; collection, management, analysis, and interpretation of data; writing of the report; and the decision to submit the report for publication, including whether they will have ultimate authority over any of these activities	Page 18, lines 1-2
	5d	Composition, roles, and responsibilities of the coordinating centre, steering committee, endpoint adjudication committee, data management team, and other individuals or groups overseeing the trial, if applicable (see Item 21a for data monitoring committee)	Page 16, lines 2-6
**Introduction**			

For peer review only - http://bmjopen.bmj.com/site/about/guidelines.xhtml

Background and rationale	6a	Description of research question and justification for undertaking the trial, including summary of relevant studies (published and unpublished) examining benefits and harms for each intervention	Page 4, lines 1-31 & Page 5 lines 1-3
	6b	Explanation for choice of comparators	Page 5, lines 22-28
Objectives	7	Specific objectives or hypotheses	Page 5, lines 4-19
Trial design	8	Description of trial design including type of trial (eg, parallel group, crossover, factorial, single group), allocation ratio, and framework (eg, superiority, equivalence, noninferiority, exploratory)	Page 5, lines 21 - 28
**Methods: Participants, interventions, and outcomes**			
Study setting	9	Description of study settings (eg, community clinic, academic hospital) and list of countries where data will be collected. Reference to where list of study sites can be obtained	Page 5, lines 21 – 30 & page 6 lines 1-6
Eligibility criteria	10	Inclusion and exclusion criteria for participants. If applicable, eligibility criteria for study centres and individuals who will perform the interventions (eg, surgeons, psychotherapists)	Page 6, lines 12-14 including tables 1 and 2 (page 7)
Interventions	11a	Interventions for each group with sufficient detail to allow replication, including how and when they will be administered	Page 7 lines 2-6 & page 8 lines 1-7
	11b	Criteria for discontinuing or modifying allocated interventions for a given trial participant (eg, drug dose change in response to harms, participant request, or improving/worsening disease)	Page 8 lines 8-12
	11c	Strategies to improve adherence to intervention protocols, and any procedures for monitoring adherence (eg, drug tablet return, laboratory tests)	Page 8 lines 8-12
	11d	Relevant concomitant care and interventions that are permitted or prohibited during the trial	N/A

For peer review only - http://bmjopen.bmj.com/site/about/guidelines.xhtml

Outcomes	12	Primary, secondary, and other outcomes, including the specific measurement variable (eg, systolic blood pressure), analysis metric (eg, change from baseline, final value, time to event), method of aggregation (eg, median, proportion), and time point for each outcome. Explanation of the clinical relevance of chosen efficacy and harm outcomes is strongly recommended	Page 8 lines 13-16 (including table 3) & page 9 lines 1-20
Participant timeline	13	Time schedule of enrolment, interventions (including any run-ins and washouts), assessments, and visits for participants. A schematic diagram is highly recommended (see Figure)	Page 9 lines 21 – 25 & page 10 lines 1-9
Sample size	14	Estimated number of participants needed to achieve study objectives and how it was determined, including clinical and statistical assumptions supporting any sample size calculations	Page 10 lines 10-18
Recruitment	15	Strategies for achieving adequate participant enrolment to reach target sample size	Page 10 lines 19-30 & page 11 lines 1-32

Methods: Assignment of interventions (for controlled trials)

Allocation:

Sequence generation	16a	Method of generating the allocation sequence (eg, computer-generated random numbers), and list of any factors for stratification. To reduce predictability of a random sequence, details of any planned restriction (eg, blocking) should be provided in a separate document that is unavailable to those who enrol participants or assign interventions	Page 12 lines 1-8
Allocation concealment mechanism	16b	Mechanism of implementing the allocation sequence (eg, central telephone; sequentially numbered, opaque, sealed envelopes), describing any steps to conceal the sequence until interventions are assigned	Page 12 lines 1-8
Implementation	16c	Who will generate the allocation sequence, who will enrol participants, and who will assign participants to interventions	Page 12 lines 1-8
Blinding (masking)	17a	Who will be blinded after assignment to interventions (eg, trial participants, care providers, outcome assessors, data analysts), and how	Page 12 lines 1-8

	17b	If blinded, circumstances under which unblinding is permissible, and procedure for revealing a participant's allocated intervention during the trial	Page 12 lines 1-8
**Methods: Data collection, management, and analysis**			
Data collection methods	18a	Plans for assessment and collection of outcome, baseline, and other trial data, including any related processes to promote data quality (eg, duplicate measurements, training of assessors) and a description of study instruments (eg, questionnaires, laboratory tests) along with their reliability and validity, if known. Reference to where data collection forms can be found, if not in the protocol	Page 12 lines 11-16, 27-30 & page 13 lines 1-31 & page 14 lines 1-6
	18b	Plans to promote participant retention and complete follow-up, including list of any outcome data to be collected for participants who discontinue or deviate from intervention protocols	Page 12 lines 11-16, 27-30 & page 13 lines 1-31 & page 14 lines 1-6
Data management	19	Plans for data entry, coding, security, and storage, including any related processes to promote data quality (eg, double data entry; range checks for data values). Reference to where details of data management procedures can be found, if not in the protocol	Page 14 lines 7-12
Statistical methods	20a	Statistical methods for analysing primary and secondary outcomes. Reference to where other details of the statistical analysis plan can be found, if not in the protocol	Page 14 lines 14 – 28 & page 15 lines 16 - 20
	20b	Methods for any additional analyses (eg, subgroup and adjusted analyses)	Page 14 lines 29-30
	20c	Definition of analysis population relating to protocol non-adherence (eg, as randomised analysis), and any statistical methods to handle missing data (eg, multiple imputation)	Page 14 lines 14 – 28
**Methods: Monitoring**			
Data monitoring	21a	Composition of data monitoring committee (DMC); summary of its role and reporting structure; statement of whether it is independent from the sponsor and competing interests; and reference to where further details about its charter can be found, if not in the protocol. Alternatively, an explanation of why a DMC is not needed	Page 15, lines 11-13

	21b	Description of any interim analyses and stopping guidelines, including who will have access to these interim results and make the final decision to terminate the trial	N/A
Harms	22	Plans for collecting, assessing, reporting, and managing solicited and spontaneously reported adverse events and other unintended effects of trial interventions or trial conduct	Page 15, lines 14-19
Auditing	23	Frequency and procedures for auditing trial conduct, if any, and whether the process will be independent from investigators and the sponsor	Page 15, lines 20 - 24
**Ethics and dissemination**			
Research ethics approval	24	Plans for seeking research ethics committee/institutional review board (REC/IRB) approval	Page 2 lines 19-23
Protocol amendments	25	Plans for communicating important protocol modifications (eg, changes to eligibility criteria, outcomes, analyses) to relevant parties (eg, investigators, REC/IRBs, trial participants, trial registries, journals, regulators)	Page 16, lines 21-22
Consent or assent	26a	Who will obtain informed consent or assent from potential trial participants or authorised surrogates, and how (see Item 32)	Page 10 lines 19-30, page 11 lines 1-31
	26b	Additional consent provisions for collection and use of participant data and biological specimens in ancillary studies, if applicable	Page 10 lines 19-30, page 11 lines 1-31
Confidentialit y	27	How personal information about potential and enrolled participants will be collected, shared, and maintained in order to protect confidentiality before, during, and after the trial	Page 16, lines 24-27
Declaration of interests	28	Financial and other competing interests for principal investigators for the overall trial and each study site	Page 16 lines 28-29
Access to data	29	Statement of who will have access to the final trial dataset, and disclosure of contractual agreements that limit such access for investigators	Page 17 lines 2-5
Ancillary and post-trial care	30	Provisions, if any, for ancillary and post-trial care, and for compensation to those who suffer harm from trial participation	N/A

For peer review only - http://bmjopen.bmj.com/site/about/guidelines.xhtml

Dissemination policy	31a	Plans for investigators and sponsor to communicate trial results to participants, healthcare professionals, the public, and other relevant groups (eg, via publication, reporting in results databases, or other data sharing arrangements), including any publication restrictions	Page 17, lines 8 -17
	31b	Authorship eligibility guidelines and any intended use of professional writers	Page 17 lines 19-23
	31c	Plans, if any, for granting public access to the full protocol, participant-level dataset, and statistical code	N/A
**Appendices**			
Informed consent materials	32	Model consent form and other related documentation given to participants and authorised surrogates	N/A
Biological specimens	33	Plans for collection, laboratory evaluation, and storage of biological specimens for genetic or molecular analysis in the current trial and for future use in ancillary studies, if applicable	N/A

*It is strongly recommended that this checklist be read in conjunction with the SPIRIT 2013 Explanation & Elaboration for important clarification on the items. Amendments to the protocol should be tracked and dated. The SPIRIT checklist is copyrighted by the SPIRIT Group under the Creative Commons "Attribution-NonCommercial-NoDerivs 3.0 Unported" license.

For peer review only
