# Supplementary material for: The SEE-IT Trial: emergency medical services Streaming Enabled Evaluation In Trauma: study protocol for an interventional feasibility randomised controlled trial
Source: BMJ Open. 2023 Apr 24;13(4):e072877. doi: 10.1136/bmjopen-2023-072877 (PMC10151834; doi:10.1136/bmjopen-2023-072877)
Supplement: Reviewer comments [file bmjopen-2023-072877.reviewer_comments.pdf]

PEER REVIEW HISTORY

BMJ Open publishes all reviews undertaken for accepted manuscripts. Reviewers are asked to complete a checklist review form (**http://bmjopen.bmj.com/site/about/resources/checklist.pdf**) and are provided with free text boxes to elaborate on their assessment. These free text comments are reproduced below.

ARTICLE DETAILS

**TITLE (PROVISIONAL)**	The SEE-IT Trial: Emergency Medical Services Streaming Enabled Evaluation In Trauma: study protocol for an interventional feasibility randomised controlled trial
**AUTHORS**	Ollis, Lucie; Skene, Simon; Williams, Julia; Lyon, Richard; Taylor, Cath; The SEE-IT Trial Group, The SEE-IT Trial Group

This article was not externally reviewed at BMJ Open. Protocols that have been independently assessed prior to submission to BMJ Open are usually fast-tracked to publication on the grounds that further substantial changes will not be possible. This independent assessment will usually be external, independent review for both a substantial grant award from a non-commercial or government funder and ethics approval.
